# Optimizing the enzymatic release of MMAE from *iso*DGR-based small molecule drug conjugate by incorporation of a GPLG-PABC enzymatically cleavable linker

**DOI:** 10.3389/fphar.2023.1215694

**Published:** 2023-07-10

**Authors:** Marco Zambra, Ivan Ranđelović, Francesco Talarico, Adina Borbély, Laura Svajda, József Tóvári, Gábor Mező, Lizeth Bodero, Sveva Colombo, Federico Arrigoni, Elettra Fasola, Silvia Gazzola, Umberto Piarulli

**Affiliations:** ^1^ Science and High Technology Department, University of Insubria, Como, Italy; ^2^ The National Tumor Biology Laboratory, Department of Experimental Pharmacology, National Institute of Oncology, Budapest, Hungary; ^3^ MTA-ELTE Lendület Ion Mobility Mass Spectrometry Research Group and Faculty of Science, Institute of Chemistry, ELTE Eötvös Loránd University, Budapest, Hungary; ^4^ KINETO Lab Ltd., Budapest, Hungary; ^5^ Doctoral School of Pathological Sciences, Semmelweis University, Budapest, Hungary; ^6^ ELKH-ELTE Research Group of Peptide Chemistry, Faculty of Science, Eötvös Loránd University, Budapest, Hungary; ^7^ Faculty of Science, Institute of Chemistry, Eötvös Loránd University, Budapest, Hungary; ^8^ Department of Chemistry Organic and Bioorganic Chemistry, Bielefeld University, Bielefeld, Germany

**Keywords:** drug release, tumor targeting, small molecule-drug conjugate, monomethyl auristatin E, cleavable linker, drug delivery, GPLG-linker

## Abstract

Antibody-Drug Conjugates (ADCs) and Small Molecule-Drug Conjugates (SMDCs) represent successful examples of targeted drug-delivery technologies for overcoming unwanted side effects of conventional chemotherapy in cancer treatment. In both strategies, a cytotoxic payload is connected to the tumor homing moiety through a linker that releases the drug inside or in proximity of the tumor cell, and that represents a key component for the final therapeutic effect of the conjugate. Here, we show that the replacement of the Val-Ala-*p*-aminobenzyloxycarbamate linker with the Gly-Pro-Leu-Gly-*p*-aminobenzyloxycarbamate (GPLG-PABC) sequence as enzymatically cleavable linker in the SMDC bearing the *cyclo*[DKP*-iso*DGR] α_V_β_3_ integrin ligand as tumor homing moiety and the monomethyl auristatin E (MMAE) as cytotoxic payload led to a 4-fold more potent anti-tumoral effect of the final conjugate on different cancer cell lines. In addition, the synthesized conjugate resulted to be significantly more potent than the free MMAE when tested following the “kiss-and-run” protocol, and the relative potency were clearly consistent with the expression of the α_V_β_3_ integrin receptor in the considered cancer cell lines. *In vitro* enzymatic cleavage tests showed that the GPLG-PABC linker is cleaved by lysosomal enzymes, and that the released drug is observable already after 15 min of incubation. Although additional data are needed to fully characterize the releasing capacity of GPLG-PABC linker, our findings are of therapeutic significance since we are introducing an alternative to other well-established enzymatically sensitive peptide sequences that might be used in the future for generating more efficient and less toxic drug delivery systems.

## 1 Introduction

**FIGURE 1:**
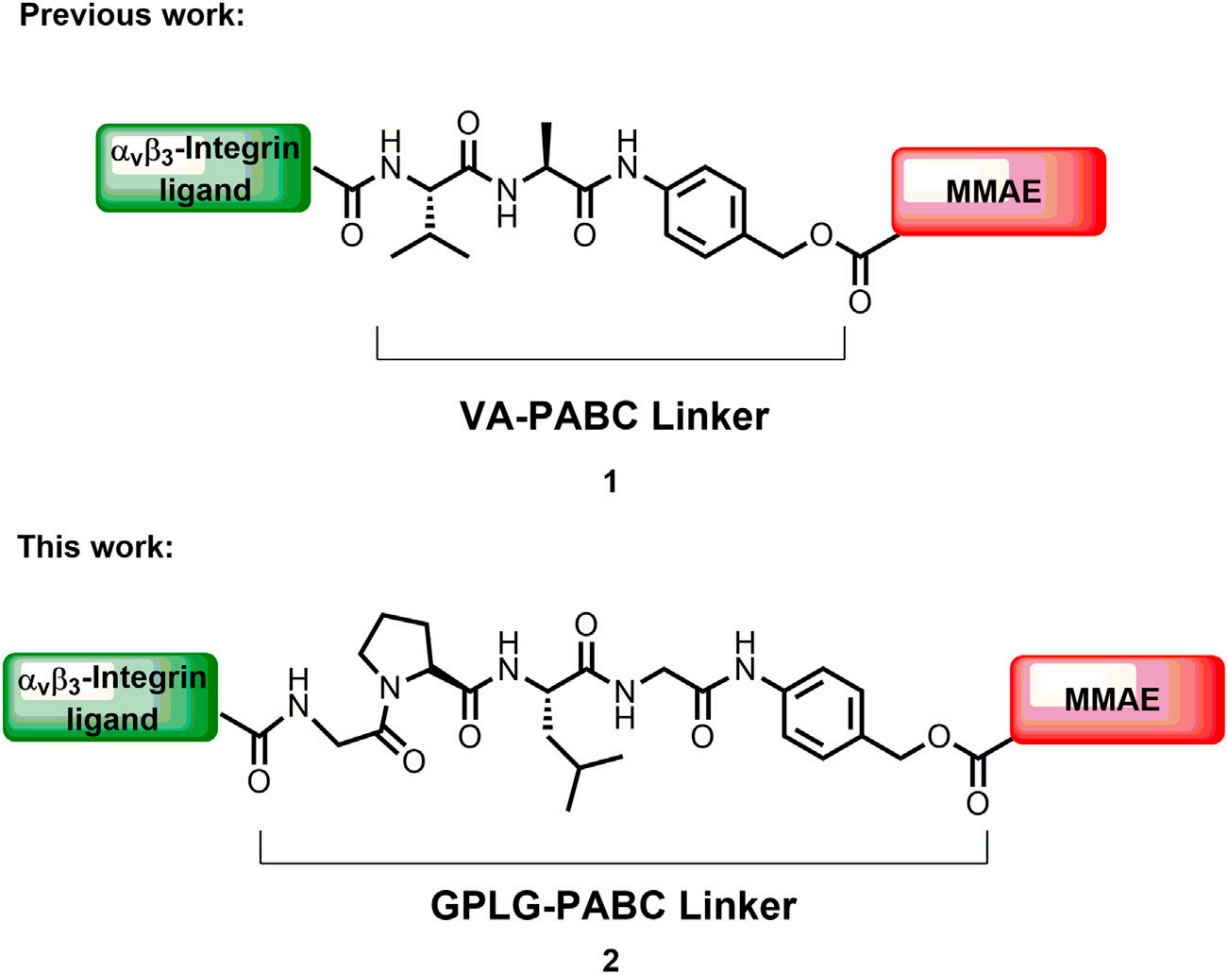
The MMAE-based SMDC previously synthesized by us (**1**), and the MMAE-based SMDC of this work (**2**).

Over the last 2 decades, the use of targeted drug-delivery technologies has received a remarkable attention for overcoming unwanted side effects of conventional chemotherapy (Ashley, 2016; Su et al., 2019; Roberts et al., 2020). Antibody-Drug Conjugates (ADCs) represent successful examples of targeting technology (Park et al., 2022) that can selectively deliver a highly potent toxin to a cancer cell thanks to the high affinity of the antibody towards a specific cell surface antigen. The general structure of ADCs includes a monoclonal antibody (mAb) connected to a therapeutic payload by a linker that ideally should facilitate the release of the cytotoxic payload inside or in proximity of the tumor cell (Srinivasarao et al., 2015; Dal Corso et al., 2017a; Srinivasarao and Low, 2017; Cazzamalli et al., 2018; Lambert and Berkenblit, 2018). Although fourteen ADCs have been approved by FDA (Chia, 2022; Fu et al., 2022), and more than 100 are in clinical/preclinical trials at present, several drawbacks deriving by the use of mAbs like immunogenicity and poor pharmacokinetics, still limit their use (Krall et al., 2013), and new drug delivery systems (DDSs) are requested. Small Molecule-Drug Conjugate (SMDCs) constitute an alternative emerging strategy to direct a cytotoxic payload to cancer cells (Zhuang et al., 2019). In these constructs, the mAb is replaced by a small molecule that binds with high affinity (Kd ≥ of 10 nM) a specific cell surface antigen. Their easier synthesis, the accurate toxin to ligand ratio (compared to the drug to antibody ratio, DAR) and the lower molecular weight that increases cell permeability and decreases accumulation in healthy organs, are the main features that make SMDCs an attractive research field. Folic acid derivatives are considered the first small molecule ligands used for the selective delivery of cytotoxic payloads to tumors overexpressing folate receptor (Reddy et al., 2020). In particular, the compound known as vintafolide (or EC145), in which the folic acid ligand is connected to desacetylvinblastine through a disulfide bond, is in phase III clinical trials (ID: NCT01170650). To follow, prostate specific membrane antigen (Olatunji et al., 2022), somatostatin receptor (Figueras et al., 2019), glucose transporter 1 receptor (Fu et al., 2020) and α_V_β_3_ integrin receptor (Lerchen et al., 2022) ligands have been also efficiently used in SMDCs that are currently in preclinical or clinical development (Patel et al., 2021). Furthermore, two radioactive SMDCs (Hennrich and Kopka, 2019; Hennrich and Eder, 2022) were recently approved by FDA and EMA and are now commercially available. In these SMDCs, the most used cleavable linkers to connect the small ligand to the cytotoxic payload are: disulfide bonds, ester and amide functionalities, and maleimido-moiety bound to self-immolative portions, followed by dipeptide sequences such as valine-alanine (VA) or valine-citrulline (VCit) that can be cleaved by tumor overexpressed proteases (Zhuang et al., 2019; Patel et al., 2021). In the last years, our group has been working on the development of SMDCs based on α_V_β_3_ integrin ligands as targeting device for cancer cells. Integrins are transmembrane receptors that are over-expressed on the cell surface of several tumors like glioblastoma (Gladson and Cheresh, 1991), melanoma (Brooks et al., 1994), prostate (Heß et al., 2014), pancreatic cancer (Hosotani et al., 2002) and others, and are involved in several biological processes linked to the tumor growth. To this aim, we designed a cyclic peptidomimetic containing a diketopiperazine (DKP) and the α_v_β_3_ integrin recognizing sequence Arg-Gly-Asp (RGD) (Marchini et al., 2012; da Ressurreição et al., 2009) or *iso*Asp-Gly-Arg (*iso*DGR) (Mingozzi et al., 2013; Panzeri et al., 2015) with low-nanomolar affinity, which were conjugated to different cytotoxic payloads such as α-amanitin (Bodero et al., 2018), paclitaxel (PTX) (Dal Corso et al., 2015; Dias et al., 2017; Rivas et al., 2018; Bodero et al., 2021), and auristatin derivatives (Dias et al., 2019) through the cathepsin-sensitive VA linker. In addition, the self-immolative spacer *p*-aminobenzyloxycarbamate (PABC) was inserted to facilitate the release of the drug upon enzymatic cleavage. The resulting SMDCs showed effectively high binding affinity and excellent selectivity towards α_V_β_3_ integrin, but a marked loss of potency compared to the free drugs was always observed, probably caused by a poor integrin-mediated internalization process upon ligand binding (Sancey et al., 2009; Kemker et al., 2020). As example, the recently reported SMDC **1** formed by the α_V_β_3_ integrin ligand *cyclo*[DKP-*iso*DGR] peptidomimetic, the potent cytotoxic agent monomethyl auristatin E (MMAE) and the VA cleavable linker, showed an interesting low nanomolar IC_50_ when tested on U87MG human glioblastoma cancer cell lines. However, a remarkable loss of potency of two orders of magnitude of the conjugate was observed compared to the free drug, which could be attributed to a non-efficient integrin-mediated internalization or to a poor release of the free drug from the enzymatically cleavable linker (Dias et al., 2019). A common strategy to improve the efficacy of ADCs and SMDCs relies on the optimization of the release mechanism of the payload from the conjugate. Thus, in our continue research of optimizing integrin ligand-based SMDCs, we report herein a novel promising SMDC (2, Figure 1), in which the potent payload monomethyl auristatin E (MMAE) is conjugated to the *cyclo*[DKP-*iso*DGR] α_V_β_3_ integrin ligand through the enzymatically cleavable linker Gly-Pro-Leu-Gly-*p*-aminobenzyl carbamate (GPLG-PABC).

GPLG is one of the recognition sequences of the matrix metalloproteinase 2 (MMP-2), which is co-localized on the cell surface with the integrin α_V_β_3_ receptor (Brooks et al., 1996) and is overexpressed in tumors, such as glioblastoma and malignant melanoma. Sequences of this kind (*i.e.*, GPLG-LAGDD) have been sparely used in ADCs and SMDCs as extracellular cleavable linkers (Eckhard et al., 2016; Poreba, 2020) in the case of non-internalizing mAbs or ligands, respectively. Since the MMP-2 protease cleavage site P-P′ is located between the Gly (P) and a further hydrophobic amino acid residue such as Leu or Val (P′) (Eckhard et al., 2016), the enzymatic release of the payload generates a functionalized drug that often displays a lower cytotoxicity, thus limiting their use in the targeted drug delivery field. This is the case of the MMP-2 sensitive peptide-doxorubicin conjugates reported by Lee and coworkers: the HPLC trace obtained after incubation of the conjugates with active MMP-2 did not show peaks corresponding to the free doxorubicin, and they concluded that only peptide-doxorubicin fragments are released as degradation products (Lee et al., 2006). MMAE needs to be released fully underivatized for exploiting its full cytotoxic potency (Doronina et al., 2003), and for this reason we directly linked the GPLG sequence to the aromatic amino group of the self-immolative linker PABC to obtain the free drug upon enzymatic release (**2**, Figure 1). We indeed hypothesized that the presence of the hydrophobic PABC moiety could mimic the hydrophobic residue normally present at the P’ position favoring its recognition by the proteolytic enzyme. The conjugate **2** was synthesized and tested on three positive α_V_β_3_ integrin overexpressing human cancer cell lines (U87MG Malignant Glioblastoma, SK-MEL-28 Melanoma and SK-OV-3 Ovarian Cancer), and on the negative α_V_β_3_ integrin A549 human lung cancer cell line. The cytotoxicity and the targeting ability were evaluated by antiproliferative assay with two different experimental setups, and the drug release mechanism of the GPLG-PABC linker was then investigated by additional antiproliferative and *in vitro* enzymatic cleavage tests. The obtained data indicated that the *in vitro* release of MMAE from the GPLG-PABC system is efficient, and that is surprisingly triggered by lysosomal enzymes rather than MMP-2 protease. Our results introduce a new lysosomal cleavable linker with promising features for generating improved ADCs and SMDCs for therapeutic uses.

## 2 Materials and methods

### 2.1 Chemistry: general information

All commercially available reagents were purchased from Sigma-Aldrich, Fluorochem, Tokyo Chemical Industries, Alfa Aesar, Carlo Erba, VWR Chemicals and used as received without further purifications. All manipulations requiring anhydrous conditions were carried out in flame-dried glassware with magnetic stirring under nitrogen atmosphere. Anhydrous solvents were withdrawn from the container by syringe, under a slight positive pressure of nitrogen. Reactions were checked by analytical thin-layer chromatography (TLC) using silica gel pre-coated ALUGRAM Xtra SIL G/UV_254_ plates (0.60 mm thickness) purchased from Macherey-Nagel. TLCs were examined by visualization under UV light (*λ* = 254 nm) and/or staining with ninhydrin, ceric ammonium-molybdate or potassium permanganate alkaline solutions. Details of the synthetic procedures can be found in the Supplementary Material S1 document. Purifications by flash-chromatography were performed using 60 Å, 230–400 mesh, 40–63 μm silica gel. Purifications by preparative HPLC were carried using a SHIMADZU LC-20AP prominence apparatus equipped with a FRC-10A fraction collector, SPD-M20A diode-array detector, CBM-20A system controller and a Sepachrom Robusta 100 Å C18 5 μm 250 × 21.2 mm column (flow 15 mL/min). All HPLC solvents were degassed for 90 min under ultrasonic treatment. Pure freeze-dried compounds were obtained from frozen aqueous solutions using a Telstar Lyo Quest −55 lyophiliser. Purities of synthetized compounds were analyzed by analytical HPLC SHIMADZU LC-20AP equipped with diode array UV detector and LiChrosorb RP-18 (5 μm) C18 column and by Waters 600 HPLC System (equipped with Phenomenex LC column 150 × 4.6 mm Synergi 4 μm Fusion RP 80 Å) coupled with MS Waters Micromass ZQ, ESI source (flow 1 mL/min). All employed HPLC-MS solvents were degassed for 20 min under a 100 mL/min helium flow. Solution ^1^H and ^13^C NMR spectra were recorded using a Bruker Avance 400 spectrometer operating respectively at 400.16 MHz and at 100.63 MHz. ^1^H and ^13^C chemical shifts are reported in ppm (δ) relative to TMS (internal standard). Coupling constants are reported in Hz and spin multiplicity is described as follow: *s* = singlet, *d* = doublet, *t* = triplet, *dd* = doublet of doublets, *m* = multiplet. High resolution mass spectra (HRMS) were obtained with Thermo Fisher Scientific Orbitrap Exploris 120 equipped with UHPLC and C18 column.

### 2.2 Peptide synthesis

Peptide sequences were prepared by manual Solid Phase Peptide Synthesis (SPPS) by the Fmoc-strategy on Fmoc-Gly preloaded Wang resin (commercially reported loading 0.4–0.8 mmol/g). The synthesis was performed using polypropylene syringes equipped with PTFE frits as reaction vessels; stirring was accomplished by a shaking plate. Each coupling step was checked through LC-MS analysis of a small-cleavage sample. Details of the synthetic procedures are reported in the Supplementary Material S1.

### 2.3 Cell lines and culture conditions

U87MG human malignant glioma, SK-MEL-28 human melanoma, SK-OV-3 human ovarian cancer, and A549 human lung cancer cell lines were purchased from ATCC. The cells were cultured in Dulbecco’s Modified Eagle’s Medium (DMEM; Biosera, Nuaille, France), supplemented with 10% heat-inactivated Fetal Bovine Serum (FBS; Biosera), and with 1% Penicillin/Streptomycin (Biosera). Cells were cultured in sterile T75 flasks with ventilation cap (Sarstedt, Nümbrecht, Germany) at 37 °C in a humidified atmosphere with 5% CO_2_ in ESCO CelCulture Incubator (ESCO, Friedberg, Germany). Manipulations with the cells were performed in biosafety cabinet (laminar) ESCO Sentinel Gold class II model AC2-4E8 (ESCO).

### 2.4 Integrin α_V_β_3_ receptor cell surface expression level determination by flow cytometry

Cells were harvested using Accutase (Sigma Aldrich, St. Louis, MO, United States), and one million cells from each cell line were used for measuring the level of integrin α_V_β_3_ receptor on the cell surface. The cells were fixed with 4% paraformaldehyde (PFA) for 10 min at room temperature (rt), washed with phosphate buffered saline (PBS, Biosera) and exposed to 3% Bovine Serum Albumin (BSA; Sigma Aldrich) in PBS for 20 min at rt. Afterwards, integrin α_V_β_3_ antibody (Anti-Integrin α_V_β_3_ antibody, clone LM609, mouse; MAB 1976, Merck) was used in a concentration of 3 µg/million cells, diluted in PBS and 3% BSA solution and incubated for 2 h at rt. A fluorescent secondary antibody was used for detection (Cell Signaling Technology, AlexaFluor^®^ 488-conjugated anti-mouse IgG Fab fragment, CST 4408, 1:1000) and incubated at rt for 30 min. As control, samples only exposed to secondary antibody were used. The fluorescence was detected using the FITC-A channel of FACSVerseTM Flow Cytometer (BD Biosciences, Franklin Lakes, NJ, United States). The FCSalyzer 0.9.22-alpha free software (SourceForge, San Diego, CA, United States) was applied to determine and evaluate gate percentage, mean fluorescence intensity (MFI) and median fluorescence intensity (MDFI).

### 2.5 *In vitro* antiproliferative activity of the conjugate, free ligand, and free drug

After standard trypsinization and harvesting cells by trypsin (Biosera), ethylenediaminetetraacetic acid (EDTA; Biosera) and PBS solution, 6×10^3^ cells per well were seeded in serum containing (10%) growth medium to 96-well plates with flat bottom (Eppendorf, Hamburg, Germany), in a 100 μL volume per well, and incubated at 37°C.

After 24 h, cells were treated with 100 µL of 9 different concentrations of the conjugate **2**, the free ligand and free drug MMAE (1.19 p.m.–20 μM, final volume in the well was 200 µL), dissolved in serum free medium and incubated for 72 h continuously (5% serum final) in the no-wash out experiment. In the wash out experiment, cells were incubated with the compounds (5% serum final) for 30 min, then they were washed out, and additionally incubated in growth medium (5% serum final) up to 72 h. The control wells were treated only with serum free medium (5% serum final).

For the evaluation of the *in vitro* antiproliferative activity of compounds, the cell viability was determined by MTT assay (3-(4,5-dimethylthiazol-2-yl)-2,5-diphenyl-tetrazolium bromide) obtained from Duchefa Biochemie (Haarlem, Netherlands).

22 µL of MTT solution (5 mg/mL in PBS, 0.5 mg/mL final) was added to each well and after 2 h incubation at 37°C, the supernatant was removed. The precipitated purple formazan crystals were dissolved in 100 µL of a 1:1 solution of dimethylsulfoxide (DMSO; Merck, St. Louis, MO, United States): 96% Ethanol (Molar Chemicals Kft., Halásztelek, Hungary) and the absorbance was measured after 15 min at λ = 570 nm by using microplate reader CLARIOstar Plus (BMG Labtech, Ortenberg, Germany). Average background absorbance (DMSO-Ethanol) was subtracted from absorbance values of control and treated wells, and cell viability was determined relative to untreated (control) wells where cell viability was arbitrarily set to 100%. Absorbance values of treated samples were normalized versus untreated control samples and interpolated by nonlinear regression analysis with GraphPad Prism 6 software (GraphPad, La Jolla, San Diego, CA, United States) to generate sigmoidal dose-response curves from which the 50% inhibitory concentration (IC_50_) values of the compounds were calculated and presented as nanomolar (nM) units. The experiments were done in triplicate and each experiment was repeated three times.

### 2.6 *In vitro* antiproliferative activity in the presence of MMP-2 inhibitor

The conjugate **2** and the free MMAE were tested in presence of the MMP-2 inhibitor *cis*-9-octadecenoyl-*N*-hydroxylamine (Merck) dissolved in DMSO (You et al., 2018). The three α_V_β_3_ integrin and MMP-2 overexpressing cancer cell lines (U87MG, SK-MEL-28, SK-OV-38) were seeded with serum containing medium (Ellijimi et al., 2018; Shim et al., 2019) and, after 8 h, were washed-out. Then, 100 µL per well of MMP-2 inhibitor (10 µM final concentration) were added and cells were incubated for 16 h, prior to the treatment. Later, the conjugate **2** and free MMAE were dissolved in 10% serum containing medium and added to reach the same concentrations evaluated in the previous test in a final volume of 100 µL per well and incubated (5% serum final) continuously for 72 h of the treatment. The experiments were performed in triplicate.

### 2.7 Enzymatic-cleavage assay with MMP-2 enzyme

The cleavage test with the MMP-2 enzyme was performed using recombinant human MMP-2 protein (Active) purchased from Abcam (ab81550). A buffer solution Tris∙HCl (50 mM), NaCl (0.2 M), CaCl_2_ (10 mM), Brij-35 (0.05%), ZnSO_4_ (50 µM), pH 7.4 was prepared in accordance with the literature (Tauro et al., 2008). A solution of EDTA (50 mM) was prepared for stopping the MMP-2 activity. For the assay, the MMP-2 enzyme (10 µg) was diluted with 100 µL Milli-Q Water, accordingly to the data sheet. A solution of the H_2_N-GPLG-PABC-MMAE **7** was prepared (0.105 mg in 0.881 mL of buffer solution). To start the assay, 25 µL MMP-2 enzyme was added to 475 µL of the linker solution to have 1:20 (w/w) enzyme: linker ratio. The final solution was stirred and incubated at 37 °C. 100 μL-aliquots were removed at 0, 6 and 24 h, and the enzymatic activity was quenched by adding 350 µL EDTA solution. The resulting solution was diluted with 180 µL Milli-Q Water and 70 µL of acetonitrile (MeCN) and analyzed by analytical HPLC SHIMADZU LC-20AP equipped with diode array UV detector and LiChrosorb RP-18 (5 μm) C18 column. A linear gradient elution was used from 10% to 90% B in 30 min (eluent A: H_2_O, 0.1% trifluoroacetic acid, TFA; eluent B: MeCN) at a flow rate of 1 mL/min and the column temperature was set to 30 °C (*vide* Supplementary Material S5). Based on previous literature data (Butowska et al., 2020), the peptide Fmoc-GPLGLAGG-OH was used as positive control for the evaluation of the MMP-2 enzymatical activity, and it was synthesized by SPPS following the general procedure reported in the Supplementary Material S1. For this, 25 µL of MMP-2 enzyme solution was added to 475 µL of Fmoc-GPLGLAGG-OH solution (0.118 mg in 1.3 mL buffer solution), and the assay was carried out with the same procedure. To establish the release of the payload, the HPLC trace of free MMAE was used for comparison.

### 2.8 Enzymatic cleavage assay with lysosomal homogenate

The enzymatically cleavage test was performed by incubating the conjugate **2** with rat liver lysosomal homogenate and analyzed by LC–MS. In particular, the conjugate **2** (2.35 μg/μL in DMSO, 5 μL) was diluted with 0.2 M NaOAc solution (pH 5.05, 495 μL) to 0.024 μg/μL. The lysosomal homogenate was prepared from rat liver (Gomena et al., 2023), and contained proteins in 71.76 μg/μL concentration. 8.6 μL of this lysosomal homogenate was diluted with 66.4 μL 0.2 M NaOAc solution (pH 5.05), to have a protein concentration of 8.27 μg/μL. To start the assay, 15 μL lysosome homogenate (8.27 μg/μL) was added to 500 μL conjugate solution (0.025 μg/μL), to have a conjugate: lysosomal protein = 1:10 (w/w) ratio. Furthermore, a control reaction mixture was prepared, containing the conjugate (2.35 μg/μL in DMSO, 5 μL) and 510 μL of 0.2 M NaOAc solution (pH 5.05). The samples were stirred at 300 rpm, 37°C and 50 µL aliquots were taken out at 0 min, 15 min, 30 min, 1 h, 2 h, 6 h, 24 h, and 72 h. The enzymatic activity was quenched by adding 5 μL formic acid (FA) to the samples. After this procedure, samples were frozen immediately at −25°C. Control samples were collected at 0 min, 1 h, 6 h, 24 h and 72 h. The composition of the samples was determined by HPLC-MS. The LC-MS analysis was carried out on an UltiMate 3000 UHPLC system (Thermo Fisher Scientific, Bremen, Germany) coupled to a Q Exactive^TM^ Focus, high resolution and high mass accuracy, hybrid quadrupole-orbitrap mass spectrometer (Thermo Fisher Scientific, Bremen, Germany). Compounds were separated on a Supelco Ascentis C18 column (150 × 2.1 mm, 3 µm) (Hesperia, CA) using a linear gradient elution from 2% to 90% B in 16 min (eluent A: H_2_O, 0.1% FA; eluent B: 80% MeCN, 0.1% FA) at a flow rate of 0.2 mL/min and the column temperature was set to 40°C. High-resolution mass spectra were acquired in the 200–2000 m*/z* range. LC-MS data were analyzed by XcaliburTM software (Thermo Fisher Scientific).

## 3 Results and discussion

### 3.1 Synthesis of *cyclo*[DKP-*iso*DGR]-PEG_4_-GPLG-PABC-MMAE (2)

**SCHEME 1:**
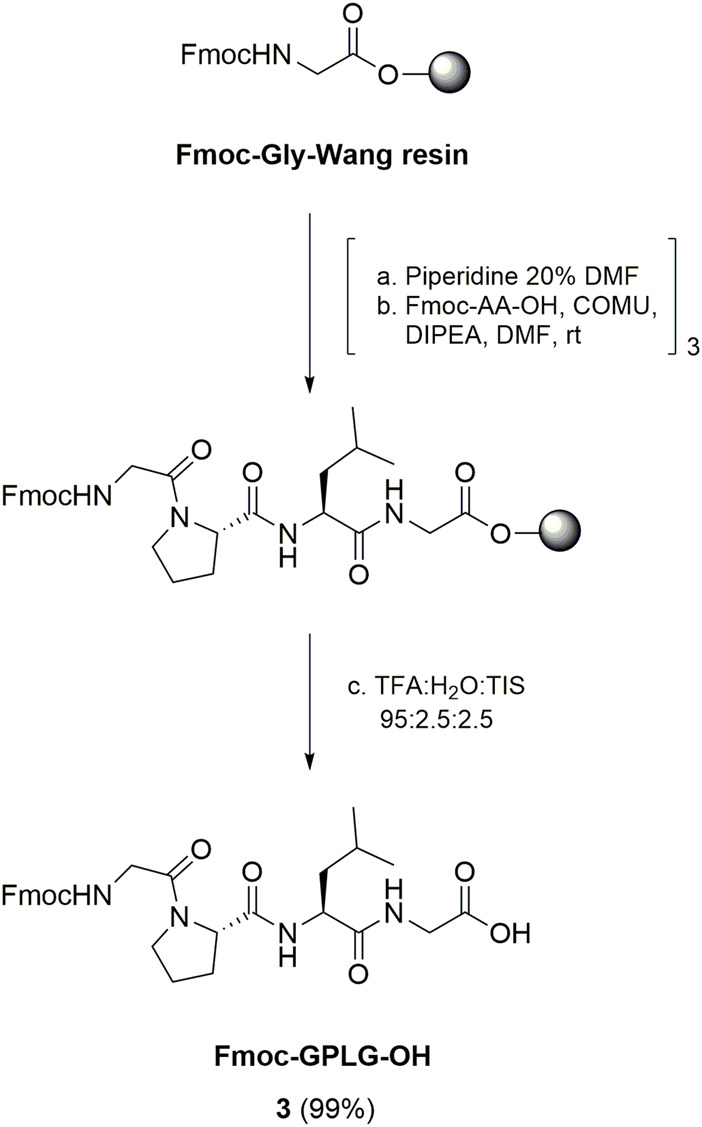
Synthesis of Fmoc-GPLG-OH **3** by SPPS.

The synthesis of the conjugate *cyclo*[DKP-*iso*DGR]-PEG_4_-GPLG-PABC-MMAE **2** started from the preparation of the GPLG peptide sequence (Scheme 1). Fmoc-GPLG-OH **3** was prepared by manual SPPS starting from Fmoc-Gly preloaded Wang resin (commercially reported loading 0.4–0.8 mmol/g), which was subsequently coupled to Fmoc-Leu (5 eq) using (1-cyano-2-ethoxy-2-oxoethylidenaminooxy)dimethylamino-morpholino-carbenium hexafluorophosphate (COMU) as coupling reagent (5 eq) and *N*,*N*-di*iso*propylethylamine (DIPEA, 8.5 eq) as base, after removal of the Fmoc-protecting group. The same procedure was used for the successive coupling reactions with Fmoc-Pro and Fmoc-Gly, respectively. After cleavage from the resin with TFA/H_2_O/tri*iso*propyl silane (TIS) 95:2.5:2.5, the peptide was obtained with quantitative yield after purification by flash-chromatography column (Scheme 1).

**SCHEME 2:**
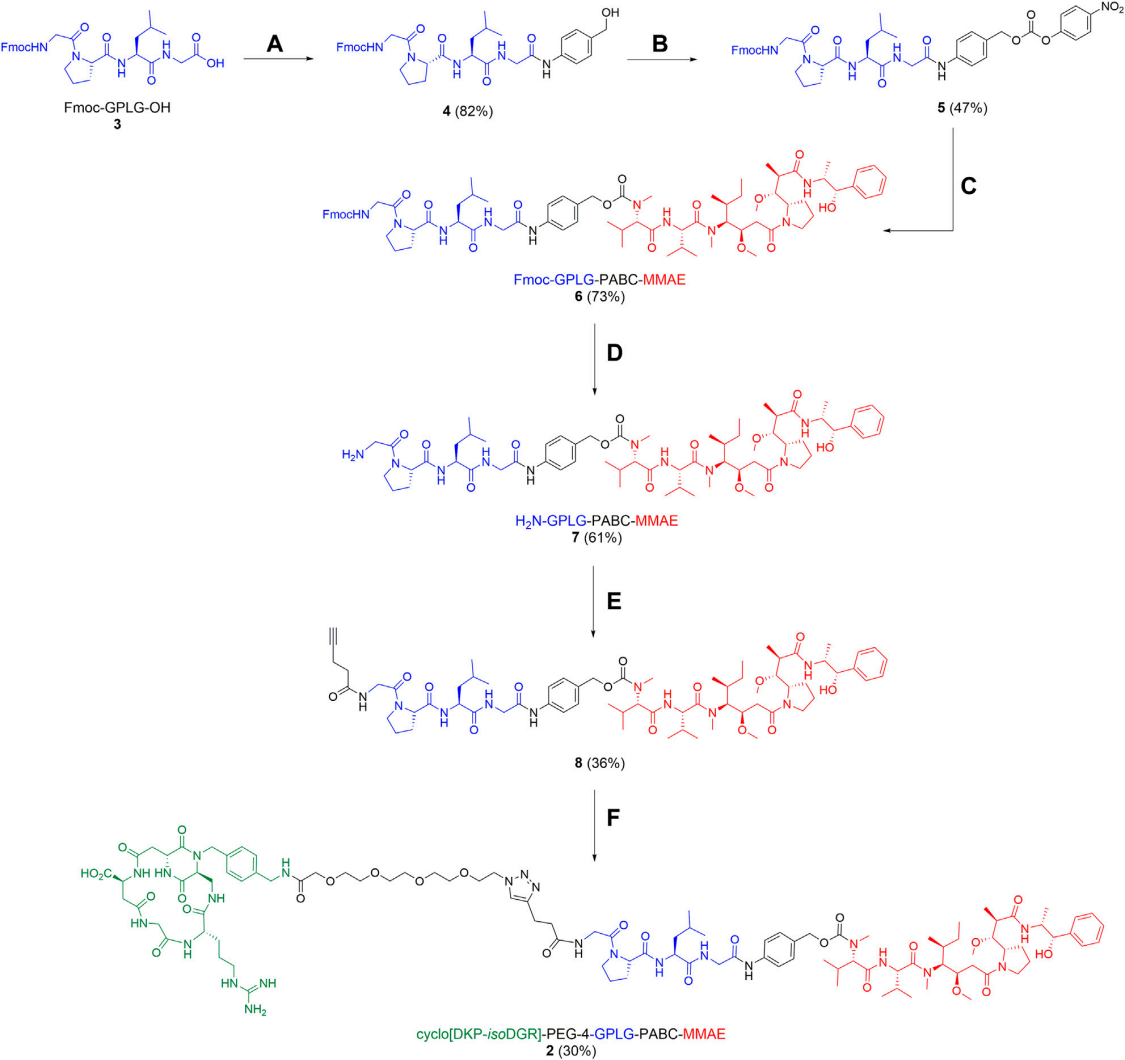
Synthesis of conjugate **2**. Reagents and conditions: **(A)** PAB-OH, EEDQ, DCM/MeOH, rt, overnight; **(B)** 4-nitrophenylchloroformate, pyridine, THF, rt, 6 h; **(C)** MMAE, HOAt, DIPEA, DMF, rt, 46 h; **(D)** piperidine, DMF, rt, 2 h; **(E)** *N*-(4-pentynoloxy)succinimide, DIPEA, DMF, rt, 2 h; **(F)** *cyclo*[DKP-*iso*DGR]-PEG_4_-N_3_ **9**, CuSO_4_∙5H_2_O, sodium ascorbate, DMF/H_2_O (1:1), rt, 25 h.

The synthesis of Fmoc-GPLG-PABC-MMAE fragment (**6**) was performed as described in Scheme 2. After treatment of Fmoc-GPLG-OH **3** with 2-ethoxy-1-ethoxycarbonyl-1,2-dihydroquinoline (EEDQ) and PAB-OH (82% yield), compound **4** was treated with 4-nitrophenyl chloroformate and pyridine in THF for 6 h, affording product **5** in 47% yield. MMAE was then conjugated to the carbonate derivative **5** using DIPEA as base and 1-hydroxy-7-azabenzotriazole (HOAt) in *N*,*N*-dimethylformamide (DMF), leading to the desired Fmoc-GPLG-PABC-MMAE (**6**) in 73% yield. Sequential Fmoc-cleavage in the presence of piperidine, which afforded the free amine **7**, and subsequent reaction with 4-pentynoic acid-NHS ester led to alkyne **8** in moderate yield (36%). A copper(I)-catalyzed azide-alkyne cycloaddition (CuAAC) «click » reaction was finally used to conjugate **8** and the functionalized ligand *cyclo*[DKP-*iso*DGR]-PEG_4_-azide **9**, prepared as previously reported (Bodero et al., 2018), which gave the SMDC **2** in 30% yield (Scheme 2).

### 3.2 *In vitro* antiproliferative activity by no-washout experiment

**FIGURE 2:**
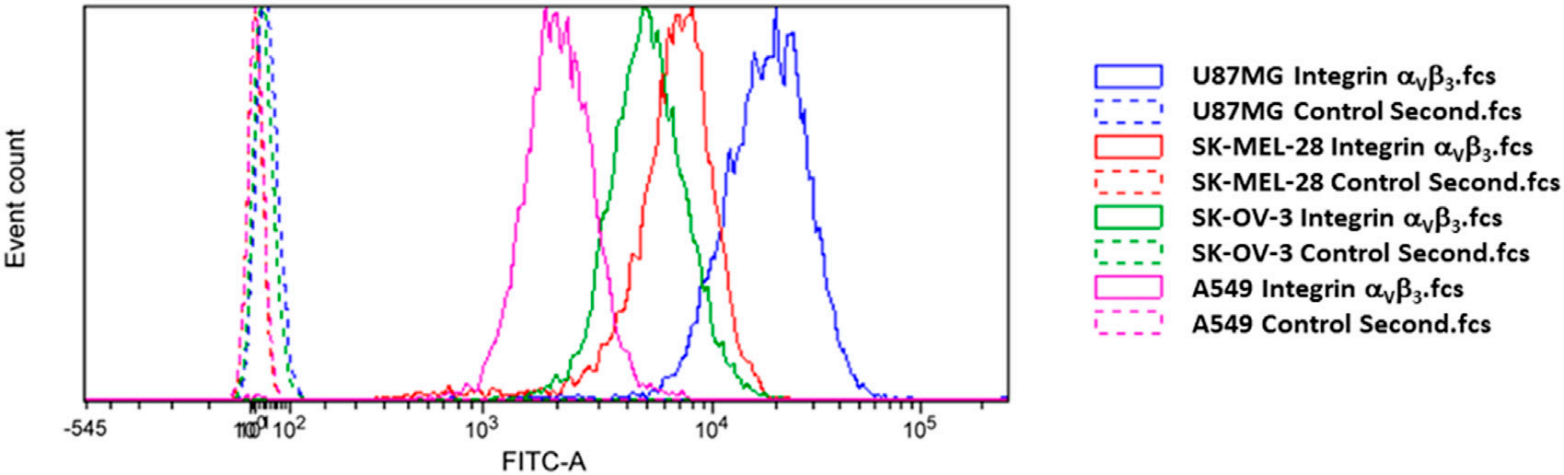
Flow cytometry analysis of integrin α_V_β_3_ receptor level of expression in the four cancer cell lines used in this work.

To evaluate the cytotoxicity of our novel SMDC **2**, we chose U87MG, SK-MEL-28, SK-OV-3 cancer cell lines, which are reported to overexpress both the α_V_β_3_ integrin receptor and the MMP-2 enzyme (Brooks et al., 1996). Additionally, we chose the A549 cancer cell line as low expressing α_V_β_3_ integrin receptor to validate the targeting. The expression of α_V_β_3_ integrin was anyway measured by flow cytometry, confirming a higher content in the case of U87MG, SK-MEL-28, SK-OV-3 compared to A549 cancer cell lines as shown in Figure 2.

**FIGURE 3:**
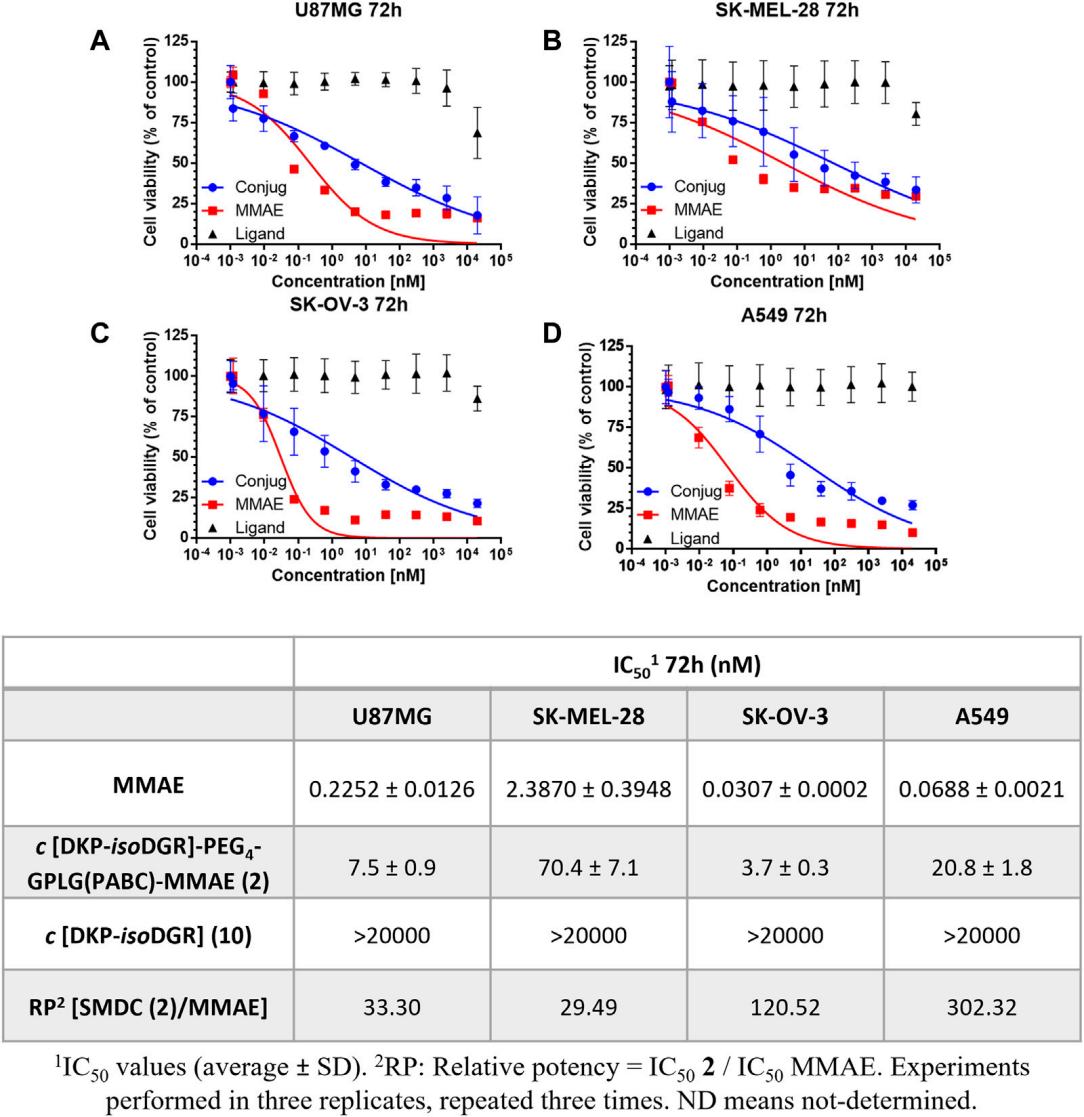
Cell viability curves and calculated IC_50_ values of cancer cell lines with different integrin α_V_β_3_ receptor level of expression after 72 h of continuous treatment with the conjugate **2**, free drug MMAE, and free ligand. **(A)** U87MG. **(B)** SK-MEL-28. **(C)** SK-OV-3. **(D)** A549.

In a first instance we performed an *in vitro* antiproliferative assay in which the different cell lines were incubated with different concentrations of the conjugate **2** for 72 h. As reported in Figure 3, **2** showed a potent antitumor activity in a low nanomolar range, from 3.7 to 70.4 nM depending on the cancer cell line, whereas the treatment with the free drug MMAE led to an antitumor activity of 0.03 nM for SK-OV-3 and of 0.23 nM for U87MG, and in very low nanomolar range on SK-MEL-28 cell line (Figure 3).

Interestingly, the relative potency (RP) of the conjugate, normalized with respect to MMAE (*i.e*., the ratio of the IC_50_ values of **2** vs*.* MMAE) in U87MG cancer cell line after 72 h was much lower than the one observed with the previously reported *cyclo*[DKP*-iso*DGR]-PEG_4_-VA-PABC-MMAE (33-fold loss of potency vs*.* 151-fold loss of potency, respectively) bearing the VA linker (Dias et al., 2019). This data could suggest a positive effect of the GPLG cleavable linker on the release of the MMAE. Furthermore, a drop of potency between the free drug and the conjugate can be noticed in A549 from the RP value, where the integrin receptor level is significantly lower than in the other cancer cell lines. To better explore the targeting ability of the conjugate **2** towards different level of integrin expression, we decided to further evaluate the antiproliferative activity of the GPLG-conjugate by changing the incubation time.

### 3.3 *In vitro* antiproliferative activity by the “kiss-and-run”-like experiment

Recently, a new protocol for evaluating the antiproliferative activity and the targeting ability of integrin-ligand based conjugates was developed by the group of Prof. Neundorf and ourselves (Feni et al., 2019). In this procedure, which we named “kiss-and-run” experiment, after very short initial contact times (15–30 min) of the toxin with the cells *in vitro*, the solution is drained, and cells are resuspended in the culture medium for a 72 h incubation. This protocol is aimed at simulating the rapid clearance of the drugs that occurs *in vivo* in the extracellular tumor environment, thus highlighting the ability of the conjugate to bind the integrin receptor on the cell surface.

**FIGURE 4:**
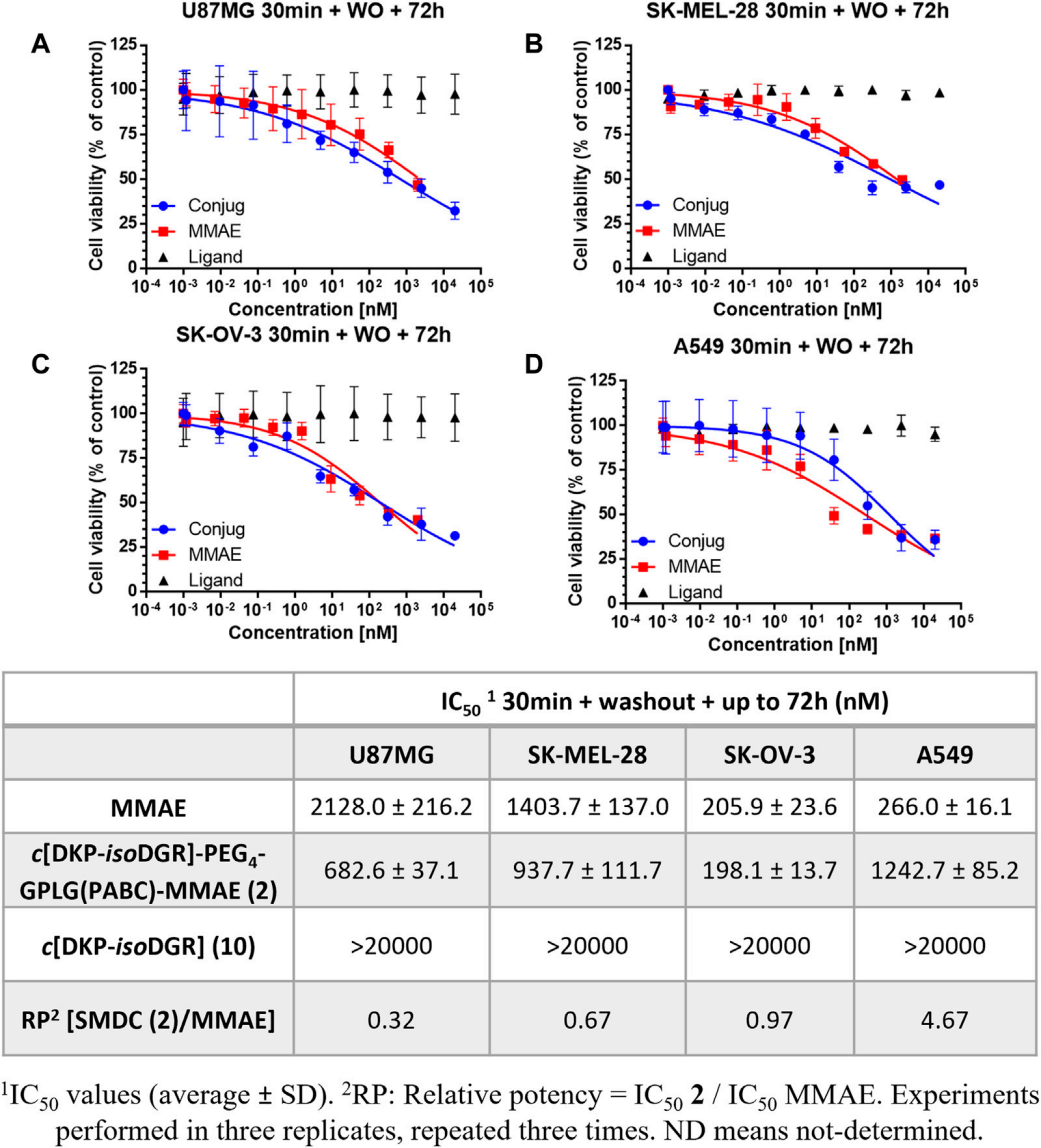
Cell viability curves and calculated IC_50_ values obtained after 30 min treatment of cancer cell lines with different integrin α_V_β_3_ receptor level of expression with the conjugate **2**, free drug MMAE, and free ligand, followed by washout and further incubation of the cells up to 72 h **(A)** U87MG. **(B)** SK-MEL-28. **(C)** SK-OV-3. **(D)** A549.

Thus, the 4 cell cultures were incubated with conjugate **2** or free MMAE for 30 min, followed by a washout of the treatment and a further incubation of the cells up to 72 h. As hypothesized, our conjugate showed higher antiproliferative activity than free MMAE on U87MG, SK-OV-3 and SK-MEL-28 integrin α_V_β_3_ receptor overexpressing cancer cell lines in comparison to low expressing cell line A549, with nanomolar activity (Figure 4).

The results clearly indicate that *cyclo*[DKP*-iso*DGR]-PEG_4_-GPLG-PABC-MMAE (**2**) is able to bind the surface of those cancer cells showing higher level of integrin overexpression (U87, SK-MEL-28 and SK-OV-3), and that the washout process does not remove it from the cell culture media. By contrast, the low level of integrin expression in A549 led to less amount of bound conjugate to the surface, and thus the washout experiment tends to eliminate it, eventually leading to a more significant decrease of the toxicity (Figure 4). Remarkably, the conjugate revealed higher antiproliferative activity compared to free drug MMAE on U87MG and SK-MEL-28, and very similar on SK-OV-3, whereas the free drug was more potent compared to the conjugate in the low integrin α_V_β_3_ receptor expressing cancer cell line A549. The relative potency (RP) of the conjugate in the different cell lines shows a good correlation with the α_V_β_3_ integrin expression. This important result confirms the efficacy of the *cyclo*[DKP*-iso*DGR] α_V_β_3_ integrin ligand in retaining the SMDC thus highlighting its targeting role. The final issue to be confirmed was whether the drug release mechanism relied on the extracellular enzymatic cleavage of the GPLG linker by the MMP-2, and subsequent internalization of MMAE by simple passive diffusion. To better elucidate the MMP-2 ability to recognize and cleave the linker, we performed a further antiproliferative bioassay adding a commercially available MMP-2 inhibitor to the cell culture in the presence of the conjugate **2**.

### 3.4 GPLG-PABC is not an MMP-2 substrate

To determine whether release of the MMAE from the conjugate depends on the action of MMP-2 enzyme by cleaving the GPLG-linker, the conjugate **2** was incubated with the α_V_β_3_ integrin positive cancer cell lines in the presence of the MMP-2-inhibitor *cis*-9-octadecenoyl-*N*-hydroxylamide. This strategy has been already reported by You and co-workers to evaluate the influence of an MMP-2 cleavable linker in the observed cytotoxicity (You et al., 2018). Surprisingly, the IC_50_ values obtained after 72 h no-washout experiments were 7.4 nM, 28.7 nM and 3.2 nM for U87MG, SK-MEL-28, SK-OV-3, respectively (Supplementary Table S4.1), which are comparable to those obtained without adding any MMP-2 inhibitor to the cell cultures (Figure 3).

These data were further confirmed by the enzymatic test performed incubating the H_2_N-GPLG-PABC-MMAE (**7**) with the active form of MMP-2 enzyme and analyzing the final solution by HPLC. Indeed, by comparison of the HPLC retention time of **7** and the free MMAE, the latter was not observed even after 74 h of incubation at 37°C (Supplementary Figure S5.3).

The lack of MMAE release upon incubation with MMP-2 enzyme, as well as the lack of significant differences in cytotoxicity when an MMP-2 inhibitor was added into the cell media, clearly indicate that the GPLG-PABC linker used in **2** is not sensitive to the extracellular MMP-2 enzyme. Although our GPLG-PABC linker is sharing a Gly at P1 and a Pro at P3 position with the most commonly used MMP-2 recognized sequences (GPLG-IAGQ, PLG-LAG and GPVG-LIGK, where hyphen indicates the cleavage site), the PABC moiety is probably not able to replace the interactions within the active site of the enzyme usually generated by the branched amino acid at the P’ position and, more in general, by the longer aminoacidic sequence (Poreba, 2020). Thus, the linker is not recognized by the MMP-2 and consequently cannot be hydrolyzed. Nevertheless, the increase in potency observed in the non-washout experiment compared with the VA-based conjugate, and the remarkably higher potency of **2** than the free MMAE in the washout experiment, suggest that an efficient release of the drug is occurring by the action of different proteolytic agents.

### 3.5 The GPLG-PABC linker is recognized by lysosomal enzymes

Although the poor ligand-mediated internalization of small integrin ligands is well documented (Liu et al., 2018; Kemker et al., 2020), the similarity of the GPLG sequence with the well-known lysosomal recognized GFLG sequence (Ruan et al., 2015), led us to hypothesize that the cleavage might occur by lysosomal enzymes, too. Indeed, the possibility to have an efficient payload release from a drug conjugate through the action of intracellular proteolytic enzymes has been reported also for non-internalizing mAbs. For example, Neri and co-workers reported that the use of the Cathepsin B sensitive Val-Cit linker in the non-internalizing IgG (F16)−MMAE conjugate led to a selective and highly cytotoxic compound both *in vitro* and *in vivo* experiments, whereas similar products based on non-cleavable linkers were not active (Dal Corso et al., 2017b). Similar evidences were also reported for non-internalizing SMDCs: in this context, a Carbonic Anhydrase IX (CAIX) small ligand and MMAE were successful conjugated through a series of Cathepsin B sensitive peptide sequences (Cazzamalli et al., 2017). In these cases, the cleavage of such sequences has been ascribed to the presence of the lysosomal Cathepsin B also in the tumor stroma, where is devoted to degrading extracellular matrix components (Sloane, 1990). The released drug may then accumulate in the extracellular environment and internalize into cancer cells by passive diffusion. Finally, the consequent cell death may induce the release of additional proteolytic enzyme (by-stander killing effect), which could cleave further cytotoxic payload from the non-internalizing conjugate (Li et al., 2016).

**FIGURE 5:**
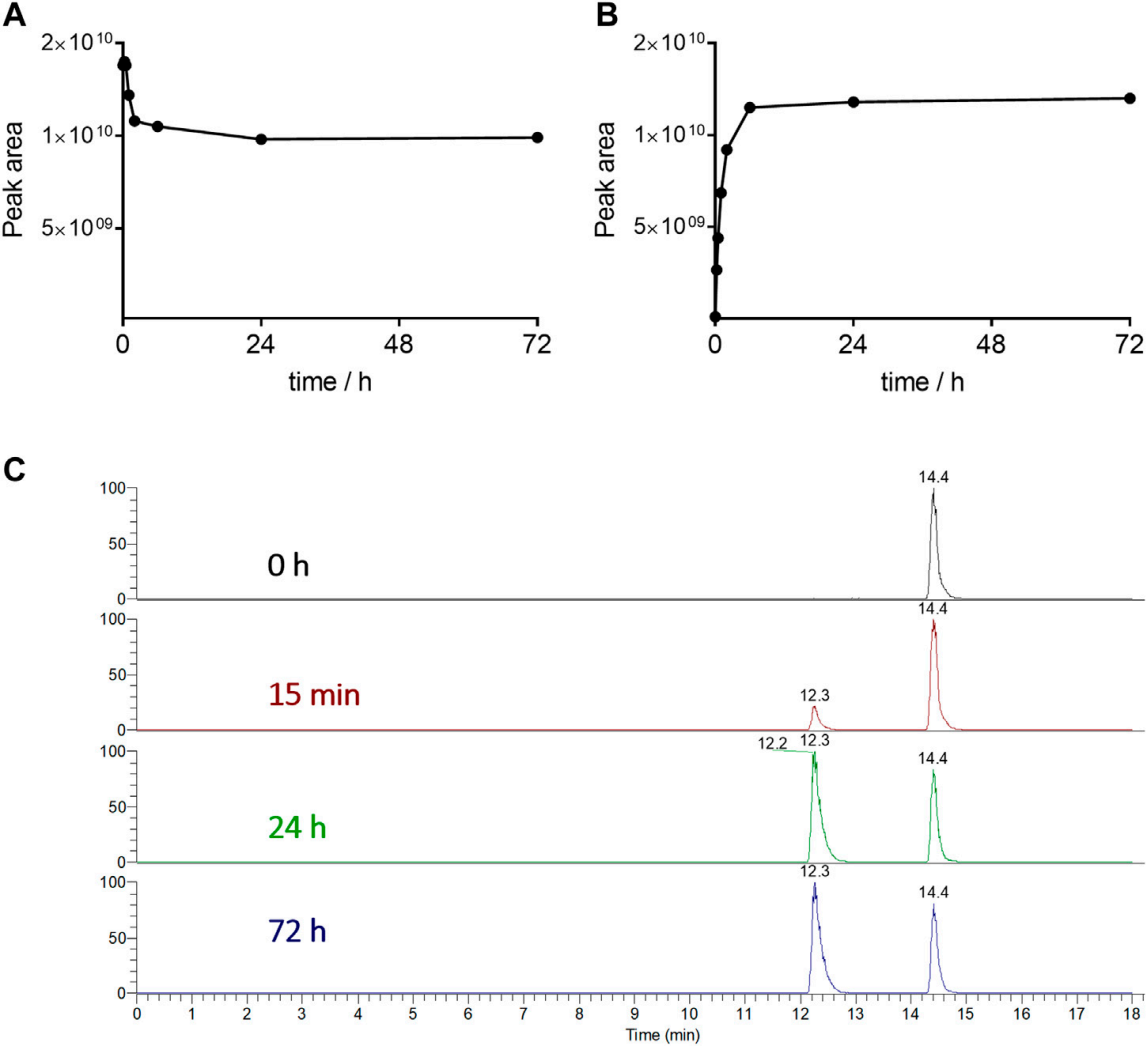
Degradation of conjugate **2 (A)** and release of the free MMAE as metabolite **(B)** in rat liver lysosomal homogenate over 72 h. Extracted Ion Chromatograms of the conjugate **2** (*t*_R_ = 14.4 min) and MMAE (*t*_R_ = 12.3 min) **(C)**.

Based on these considerations, we decided to determine the enzymatic susceptibility of conjugate **2** containing GPLG-PABC linker by studying its degradation in the presence of rat liver lysosomal homogenate. The cleavage of the conjugate, along with the release of the free MMAE was monitored by UPLC-HRMS. As shown in Figure 5C, the *cyclo*[DKP*-iso*DGR]-PEG_4_-GPLG-PABC-MMAE **2** was rapidly cleaved in the presence of lysosomal enzymes, and, upon the 1,6-elimination of the PABC spacer, the free MMAE was detectable already 15 min after incubation (Figure 5; Supplementary Figure S6.1).

Interestingly, the conjugate was not fully consumed by lysosomal enzymes even after 72 h, probably due to the decreasing activity or amount of the specific enzyme responsible for the cleavage of this specific linker type. Although further work is needed to better investigate this experimental evidence, and assuming that in the cell culture assays a similar behavior was occurring, the amount of the released MMAE was sufficient for the potent cytotoxic activity observed in the *in vitro* assays.

## 4 Conclusion

In recent years, the selective delivery of cytotoxic compounds to cancer cells using ADCs or SMDCs is gradually revolutionizing cancer therapy. In both strategies, the tumor homing moiety is connected to the cytotoxic payload through a linker, which represents a key component for the final therapeutic effect of the conjugate.

In this work, the GPLG-PABC linker was used for the first time to connect the *cyclo*[DKP*-iso*DGR] α_V_β_3_ integrin ligand (tumor homing moiety) to monomethyl auristatin E (payload). The antiproliferative activity of the final compound **2** was tested on three α_V_β_3_ integrin high expressing cancer cell lines (U87MG, SK-MEL-28, SK-OV-3) and one α_V_β_3_ integrin low expressing cancer cell line (A549). In the 72 h incubation experiment, the relative potency, normalized with respect to the free drug, showed that in U87MG cell line the *cyclo*[DKP*-iso*DGR]-PEG_4_-GPLG-PABC-MMAE conjugate is more potent than the previously synthesized VA-conjugate *cyclo*[DKP*-iso*DGR]-PEG_4_-VA-PABC-MMAE **1** (Dias et al., 2019). Additionally, the targeting ability of the conjugate **2** was consistent with the α_V_β_3_ integrin receptor level of expression on the cancer cells lines, which was obtained by flow cytometry analysis. To reinforce this positive result, the antiproliferative activity of **2** was performed following the “kiss-and-run” protocol, and the conjugate resulted to be significantly more potent than the free MMAE, with the relative potency clearly consistent with the expression of the integrin receptor in the four cancer cell lines. Finally, we demonstrated that GPLG-PABC linker is cleaved by lysosomal enzymes, and that the released drug is observable already after 15 min of incubation. Although further data are needed to fully characterize the releasing capacity of GPLG-PABC linker, the data presented in this work support the idea that a careful choice of the cleavable linker sequence is necessary to optimize the antitumor activity of SMDCs. Additionally, the use of GPLG-PABC linker in a SMDC as an efficient lysosomal cleavable linker is reported herein for the first time and, in the future, it might represent an alternative to other well-established enzymatically sensitive peptide sequences.

## Data Availability

The original contributions presented in the study are included in the article/Supplementary Material, further inquiries can be directed to the corresponding authors.

## References

[B1] Ashley E. A. (2016). Towards precision medicine. Nat. Rev. Genet. 17 (9), 507–522. 10.1038/nrg.2016.86 27528417

[B2] Bodero L. Parente S. Arrigoni F. Klimpel A. Neundorf I. Gazzola S. (2021). Synthesis and biological evaluation of an *iso*DGR-paclitaxel conjugate containing a cell-penetrating peptide to promote cellular uptake. Eur. J. Org. Chem. 2021 (17), 2383–2387. 10.1002/ejoc.202100241

[B3] Bodero L. Rivas P. L. Korsak B. Hechler T. Pahl A. Müller C. (2018). Synthesis and biological evaluation of RGD and *iso*DGR peptidomimetic-α-amanitin conjugates for tumor-targeting. Beilstein J. Org. Chem. 14 (1), 407–415. 10.3762/bjoc.14.29 29520305 PMC5827777

[B4] Brooks P. C. Clark R. A. F. Cheresh D. A. (1994). Requirement of vascular integrin α_V_β_3_ for angiogenesis. Science 264 (5158), 569–571. 10.1126/science.7512751 7512751

[B5] Brooks P. C. Strömblad S. Sanders L. C. von Schalscha T. L. Aimes R. T. Stetler-Stevenson W. G. (1996). Localization of matrix metalloproteinase MMP-2 to the surface of invasive cells by interaction with integrin alpha v beta 3. Cell 85 (5), 683–693. 10.1016/S0092-8674(00)81235-0 8646777

[B6] Butowska K. Żamojć K. Kogut M. Kozak W. Wyrzykowski D. Wiczk W. (2020). The product of matrix metalloproteinase cleavage of doxorubicin conjugate for anticancer drug delivery: Calorimetric, spectroscopic, and molecular dynamics studies on peptide–doxorubicin binding to DNA. Int. J. Mol. Sci. 21 (18), 6923. 10.3390/ijms21186923 32967212 PMC7554696

[B7] Cazzamalli S. Dal Corso A. Neri D. (2017). Linker stability influences the anti-tumor activity of acetazolamide-drug conjugates for the therapy of renal cell carcinoma. J. Control. Release 246, 39–45. 10.1016/j.jconrel.2016.11.023 27890855 PMC5266555

[B8] Cazzamalli S. Ziffels B. Widmayer F. Murer P. Pellegrini G. Pretto F. (2018). Enhanced therapeutic activity of non-internalizing small-molecule-drug conjugates targeting carbonic Anhydrase IX in combination with targeted interleukin-2. Clin. Cancer Res. 24 (15), 3656–3667. 10.1158/1078-0432.CCR-17-3457 29691298 PMC6126628

[B9] Chia C. B. (2022). A patent review on FDA-approved antibody-drug conjugates, their linkers and drug payloads. ChemMedChem 17 (11), e202200032. 10.1002/cmdc.202200032 35384350

[B10] da Ressurreição A. S. M. Vidu A. Civera M. Belvisi L. Potenza D. Manzoni L. (2009). Cyclic RGD-peptidomimetics containing bifunctional diketopiperazine scaffolds as new potent integrin ligands. Chem. Eur. J. 15 (45), 12184–12188. 10.1002/chem.200902398 19830748

[B11] Dal Corso A. Caruso M. Belvisi L. Arosio D. Piarulli U. Albanese C. (2015). Synthesis and biological evaluation of RGD peptidomimetic–paclitaxel conjugates bearing lysosomally cleavable linkers. Chem. Eur. J. 21 (18), 6921–6929. 10.1002/chem.201500158 25784522

[B12] Dal Corso A. Cazzamalli S. Gébleux R. Mattarella M. Neri D. (2017a). Protease-cleavable linkers modulate the anticancer activity of noninternalizing antibody–drug conjugates. Bioconjug. Chem. 28 (7), 1826–1833. 10.1021/acs.bioconjchem.7b00304 28662334 PMC5521252

[B13] Dal Corso A. Gébleux R. Murer P. Soltermann A. Neri D. (2017b). A non-internalizing antibody-drug conjugate based on an anthracycline payload displays potent therapeutic activity *in vivo* . J. Control. Release 264, 211–218. 10.1016/j.jconrel.2017.08.040 28867376 PMC5844458

[B14] Dias A. R. M. Bodero L. Martins A. Arosio D. Gazzola S. Belvisi L. (2019). Synthesis and biological evaluation of RGD and *iso*DGR–monomethyl auristatin conjugates targeting integrin αVβ3. ChemMedChem 14 (9), 938–942. 10.1002/cmdc.201900049 30840356 PMC6593765

[B15] Dias A. R. M. Pina A. Dal Corso A. Arosio D. Belvisi L. Pignataro L. (2017). Multivalency increases the binding strength of RGD peptidomimetic‐paclitaxel conjugates to integrin α_V_β_3_ . Chemistry 23 (58), 14410–14415. 10.1002/chem.201703093 28816404 PMC5656903

[B16] Doronina S. O. Toki B. E. Torgov M. Y. Mendelsohn B. A. Cerveny C. G. Chace D. F. (2003). Development of potent monoclonal antibody auristatin conjugates for cancer therapy. Nat. Biotechnol. 21 (7), 778–784. 10.1038/nbt832 12778055

[B17] Eckhard U. Huesgen P. F. Schilling O. Bellac C. L. Butler G. S. Cox J. H. (2016). Active site specificity profiling of the matrix metalloproteinase family: Proteomic identification of 4300 cleavage sites by nine MMPs explored with structural and synthetic peptide cleavage analyses. Matrix Biol. 49, 37–60. 10.1016/j.matbio.2015.09.003 26407638

[B18] Ellijimi C. Hammouda M. B. Othman H. Moslah W. Jebali J. Mabrouk H. B. (2018). Helix aspersa maxima mucus exhibits antimelanogenic and antitumoral effects against melanoma cells. Biomed. Pharmacother. 101, 871–880. 10.1016/j.biopha.2018.03.020 29635896

[B19] Feni L. Parente S. Robert C. Gazzola S. Arosio D. Piarulli U. (2019). Kiss and run: Promoting effective and targeted cellular uptake of a drug delivery vehicle composed of an integrin-targeting diketopiperazine peptidomimetic and a cell-penetrating peptide. Bioconjug. Chem. 30 (7), 2011–2022. 10.1021/acs.bioconjchem.9b00292 31243977

[B20] Figueras E. Martins A. Borbély A. Joncour V. L. Cordella P. Perego R. (2019). Octreotide conjugates for tumor targeting and imaging. Pharmaceutics 11 (5), 220. 10.3390/pharmaceutics11050220 31067748 PMC6571972

[B21] Fu J. Yang J. Seeberger P. H. Yin J. (2020). Glycoconjugates for glucose transporter-mediated cancer-specific targeting and treatment. Carb. Res. 498, 108195. 10.1016/j.carres.2020.108195 33220603

[B22] Fu Z. Li S. Han S. Shi C. Zhang Y. (2022). Antibody drug conjugate: The “biological missile” for targeted cancer therapy. Signal Transduct. Target. Ther. 7 (1), 93. 10.1038/s41392-022-00947-7 35318309 PMC8941077

[B23] Gladson C. L. Cheresh D. A. (1991). Glioblastoma expression of vitronectin and the alpha v beta 3 integrin. Adhesion mechanism for transformed glial cells. J. Clin. Invest. 88 (6), 1924–1932. 10.1172/jci115516 1721625 PMC295768

[B24] Gomena J. Vári B. Oláh-Szabó R. Biri-Kovács B. Bősze S. Borbély A. (2023). Targeting the gastrin-releasing peptide receptor (GRP-R) in cancer therapy: Development of bombesin-based peptide–drug conjugates. Int. J. Mol. Sci. 24, 3400. 10.3390/ijms24043400 36834815 PMC9967152

[B25] Hennrich U. Eder M. (2022). [^177^Lu]Lu-PSMA-617 (Pluvicto^TM^): The first FDA-approved radiotherapeutical for treatment of prostate cancer. Pharmaceuticals 15 (10), 1292. 10.3390/ph15101292 36297404 PMC9608311

[B26] Hennrich U. Kopka K. (2019). Lutathera®: The first FDA- and EMA-approved radiopharmaceutical for peptide receptor radionuclide therapy. Pharmaceuticals 12 (3), 114–121. 10.3390/ph12030114 31362406 PMC6789871

[B27] Heß K. Böger C. Behrens H.-M. Röcken C. (2014). Correlation between the expression of integrins in prostate cancer and clinical outcome in 1284 patients. Ann. Diagn. Pathol. 18 (6), 343–350. 10.1016/j.anndiagpath.2014.09.001 25305804

[B28] Hosotani R. Kawaguchi M. Masui T. Koshiba T. Ida J. Fujimoto K. (2002). Expression of integrin alphaVbeta3 in pancreatic carcinoma: Relation to MMP-2 activation and lymph node metastasis. Pancreas 25 (2), 30–35. 10.1097/00006676-200208000-00021 12142752

[B29] Kemker I. Feiner R. C. Müller K. M. Sewald N. (2020). Size-dependent cellular uptake of RGD peptides. ChemBioChem 21 (4), 496–499. 10.1002/cbic.201900512 31478590 PMC7064889

[B30] Krall N. Scheuermann J. Neri D. (2013). Small targeted cytotoxics: Current state and promises from DNA-encoded chemical libraries. Angew. Chem. Int. Ed. 52 (5), 1384–1402. 10.1002/anie.201204631 23296451

[B31] Lambert J. M. Berkenblit A. (2018). Antibody–drug conjugates for cancer treatment. Ann. Rev. Med. 69, 191–207. 10.1146/annurev-med-061516-121357 29414262

[B32] Lee G. Y. Song J.-h. Kim S. Y. Park K. Byun Y. (2006). Peptide-doxorubicin conjugates specifically degraded by matrix metalloproteinases expressed from tumor. Drug Dev. Res. 67, 438–447. 10.1002/ddr.20092

[B33] Lerchen H. G. Stelte-Ludwig B. Kopitz C. Heroult M. Zubov D. Willuda J. (2022). A small molecule-drug conjugate (SMDC) consisting of a modified camptothecin payload linked to an α_V_ß_3_ binder for the treatment of multiple cancer types. Cancers 14 (2), 391. 10.3390/cancers14020391 35053556 PMC8773721

[B34] Li F. Emmerton K. K. Jonas M. Zhang X. Miyamoto J. B. Setter J. R. (2016). Intracellular released payload influences potency and bystander-killing effects of antibody-drug conjugates in preclinical models. Cancer Res. 76 (9), 2710–2719. 10.1158/0008-5472.CAN-15-1795 26921341

[B35] Liu X. Wu F. Ji Y. Yin L. (2018). Recent advances in anti-cancer protein/peptide delivery. Bioconjug. Chem. 30 (2), 305–324. 10.1021/acs.bioconjchem.8b00750 30428665

[B36] Marchini M. Mingozzi M. Colombo R. Guzzetti I. Belvisi L. Vasile F. (2012). Cyclic RGD peptidomimetics containing bifunctional diketopiperazine scaffolds as new potent integrin ligands. Chemistry 18 (20), 6195–6207. 10.1002/chem.201200457 22517378

[B37] Mingozzi M. Dal Corso A. Marchini M. Guzzetti I. Civera M. Piarulli U. (2013). Cyclic *iso*DGR peptidomimetics as low‐nanomolar α_v_β_3_ integrin ligands. Chem. Eur. J. 19 (11), 3563–3567. 10.1002/chem.201204639 23424096

[B38] Olatunji F. P. Pun M. Herman J. W. Romero O. Maniatopoulos M. Latoche J. D. (2022). Modular smart molecules for PSMA-targeted chemotherapy. Mol. Cancer. Ther. 21 (11), 1701–1709. 10.1158/1535-7163.MCT-22-0160 35999662 PMC9842478

[B39] Panzeri S. Zanella S. Arosio D. Vahdati L. Dal Corso A. Pignataro L. (2015). Cyclic *iso*DGR and RGD peptidomimetics containing bifunctional diketopiperazine scaffolds are integrin antagonists. Chem. Eur. J. 21 (16), 6265–6271. 10.1002/chem.201406567 25761230

[B40] Park H. Otte A. Park K. (2022). Evolution of drug delivery systems: From 1950 to 2020 and beyond. J. Control. Release 342, 53–65. 10.1016/j.jconrel.2021.12.030 34971694 PMC8840987

[B41] Patel T. K. Adhikari N. Amin S. A. Biswas S. Jha T. Ghosh B. (2021). Small molecule drug conjugates (SMDCs): An emerging strategy for anticancer drug design and discovery. New J. Chem. 45, 5291–5321. 10.1039/D0NJ04134C

[B42] Poreba M. (2020). Protease‐activated prodrugs: Strategies, challenges, and future directions. FEBS Lett. 287 (10), 1936–1969. 10.1111/febs.15227 31991521

[B43] Reddy J. A. Nelson M. Dircksen C. Vetzel M. Johnson T. Cross V. (2020). Pre-clinical studies of EC2629, a highly potent folate-receptor-targeted DNA crosslinking agent. Sci. Rep. 10, 12772. 10.1038/s41598-020-69682-9 32728172 PMC7391724

[B44] Rivas P. L. Ranđelović I. Dias A. R. M. Pina A. Arosio D. Tovari J. (2018). Synthesis and biological evaluation of paclitaxel conjugates involving linkers cleavable by lysosomal enzymes and α_v_β_3_‐integrin ligands for tumor targeting. Eur. J. Org. Chem. 2018 (23), 2902–2909. 10.1002/ejoc.201800447

[B45] Roberts T. C. Langer R. Wood M. J. (2020). Advances in oligonucleotide drug delivery. Nat. Rev. Drug Discov. 19 (10), 673–694. 10.1038/s41573-020-0075-7 32782413 PMC7419031

[B46] Ruan H. Hao S. Young P. Zhang H. (2015). Targeting cathepsin B for cancer therapies. Horiz. Cancer Res. 56, 23–40. 26623174 PMC4662557

[B47] Sancey L. Garanger E. Foillard S. Schoehn G. Hurbin A. Albiges-Rizo C. (2009). Clustering and internalization of integrin alphavbeta3 with a tetrameric RGD-synthetic peptide. Mol. Ther. 17 (5), 837–843. 10.1038/mt.2009.29 19259068 PMC2760123

[B48] Shim G. Le Q. V. Suh J. Choi S. Kim G. Choi H. G. (2019). Sequential activation of anticancer therapy triggered by tumor microenvironment-selective imaging. J. Control. Release 298, 110–119. 10.1016/j.jconrel.2019.02.012 30771413

[B49] Sloane B. F. (1990). Cathepsin B and cystatins: Evidence for a role in cancer progression. Insemin. Cancer Biol. 1 (2), 137–152. 2103490

[B50] Srinivasarao M. Galliford C. V. Low P. S. (2015). Principles in the design of ligand-targeted cancer therapeutics and imaging agents. Nat. Rev. Drug Discov. 14 (3), 203–219. 10.1038/nrd4519 25698644

[B51] Srinivasarao M. Low P. S. (2017). Ligand-targeted drug delivery. Chem. Rev. 117 (19), 12133–12164. 10.1021/acs.chemrev.7b00013 28898067

[B52] Su H. Wang Y. Liu S. Wang Y. Liu Q. Liu G. (2019). Emerging transporter-targeted nanoparticulate drug delivery systems. Acta Pharm. Sin. B 9 (1), 49–58. 10.1016/j.apsb.2018.10.005 30766777 PMC6361857

[B53] Tauro J. R. Lee B. S. Lateef S. S. Gemeinhart R. A. (2008). Matrix metalloprotease selective peptide substrates cleavage within hydrogel matrices for cancer chemotherapy activation. Peptides 29 (11), 1965–1973. 10.1016/j.peptides.2008.06.021 18652863 PMC2592099

[B54] You Y. Xu Z. Chen Y. (2018). Doxorubicin conjugated with a trastuzumab epitope and an MMP-2 sensitive peptide linker for the treatment of HER2-positive breast cancer. Drug Deliv. 25 (1), 448–460. 10.1080/10717544.2018.1435746 29405790 PMC6058718

[B55] Zhuang C. Guan X. Ma H. Cong H. Zhang W. Miao Z. (2019). Small molecule-drug conjugates: A novel strategy for cancer-targeted treatment. Eur. J. Med. Chem. 163, 883–895. 10.1016/j.ejmech.2018.12.035 30580240

