# Supplementary material for: Optimizing the enzymatic release of MMAE from isoDGR-based small molecule drug conjugate by incorporation of a GPLG-PABC enzymatically cleavable linker
Source: Front Pharmacol. 2023 Jul 10;14:1215694. doi: 10.3389/fphar.2023.1215694 (PMC10363981; doi:10.3389/fphar.2023.1215694)
Supplement: Supplementary file 1 [file Table1.pdf]

Supplementary Material

Optimizing the Enzymatic Release of MMAE from *iso*DGR-based Small Molecule Drug Conjugate by Incorporation of a GPLG-PABC Enzymatically Cleavable Linker

Marco Zambra[1§], Ivan Ranđelović[2§], Francesco Talarico[1], Adina Borbély[3], Laura Svajda[4,5], József Tóvári[2], Gábor Mező[6,7], Lizeth Alicia Bodero[1♣], Sveva Colombo[1,8], Federico Arrigoni[1], Elettra Fasola[1], Silvia Gazzola[1*], Umberto Piarulli[1*]

[§]These two first authors have equally contributed to the work.

*** Correspondence:**

Corresponding Author: s.gazzola@uninsubria.it

Corresponding Author: umberto.piarulli@uninsubria.it

Table of Contents

S1. Synthesis 4

General procedure for solid phase peptide synthesis 4

Fmoc-GPLG-OH (**3**) 5

Fmoc-GPLGLAGG-OH 5

Fmoc-GPLG-PAB-OH (**4**) 6

Fmoc-GPLG-*N*-benzyl (4-nitrophenyl) carbonate (**5**) 7

Fmoc-GPLG-PABC-MMAE (**6**) 8

H_2N-GPLG-PABC-MMAE (**7**) 9

4-pentynoic amide-*N*-(GPLG-PABC-MMAE) (**8**) 10

cyclo[DKP-*iso*DGR]-PEG-4-GPLG-PABC-MMAE (**2**) 11

S2. TIC and MS spectra traces 12

Fmoc-GPLG-OH (**3**) 12

Fmoc-GPLGLAGG-OH 13

Fmoc-GPLG-PAB-OH (**4**) 14

Fmoc-GPLG-*N*-benzyl (4-nitrophenyl) carbonate (**5**) 15

Fmoc-GPLG-PABC-MMAE (**6**) 16

H_2N-GPLG-PABC-MMAE (**7**) 17

4-pentynoic-amide-*N*-(GPLG-PABC-MMAE) (**8**) 18

cyclo[DKP-*iso*DGR]-PEG-4-GPLG-PABC-MMAE (**2**) 19

S3. ^{1}H-NMR and ^{13}C-NMR spectra 20

Figure S3.1 ^{1}H-NMR spectrum (CD_3OD, 400 MHz) of Fmoc-GPLGLAGG-OH 20

Figure S3.2 ^{13}C-JMOD NMR spectrum (CD_3OD, 101 MHz) of Fmoc-GPLGLAGG-OH 20

Figure S3.3 ^{1}H-NMR spectrum (CD_3OD, 400 MHz) of Fmoc-GPLG-OH (**3**) 21

Figure S3.4 ^{13}C-JMOD NMR spectrum (CD_3OD, 101 MHz) of Fmoc-GPLG-OH (**3**) 21

Figure S3.5 ^{1}H NMR spectrum (Acetone-d_6, 400 MHz) of Fmoc-GPLG-PAB-OH (**4**) 22

Figure S3.6 ^{13}C-JMOD NMR spectrum (Acetone-d_6, 101 MHz) of Fmoc-GPLG-PAB-OH (**4**) 22

S4. Antiproliferative assay 23

S5. MMP-2 Degradation Assay 23

HPLC conditions for the MMP-2 cleavage studies 23

Figure S5.1 H_2N-GPLG-PABC-MMAE (**7**) + buffer 24

Figure S5.2 MMAE + buffer 24

Figure S5.3 H_2N-GPLG-PABC-MMAE (**7**): MMP-2 enzymatic cleavage assay 25

Figure S5.4 Fmoc-GPLGLAGG-OH + buffer 25

Figure S5.5 Fmoc-GPLG-OH (**3**) + buffer 26
Figure S5.6 Fmoc-GPLGLAGG-OH: MMP-2 enzymatic cleavage assay 26
S6 Lysosomal Degradation assay 27
Figure S6.1 HPLC traces from the rat-liver lysosomal homogenate degradation assay 27
Figure S6.2 HRMS spectrum of the conjugate **2**. 27
Figure S6.3 HRMS spectrum of the free MMAE. 28
Table S6.1 Analytical details of the detected analytes 28

S1. Synthesis

General procedure for solid phase peptide synthesis

Fmoc-GPLG-OH (**3**) and Fmoc-GPLGLAGG-OH as control for the MMP2 enzymatically cleavage test, were synthetized manually on the commercially available Fmoc-Gly-Wang resin (loading: 0.4 – 0.8 mmol/g) using the Fmoc protocol. This is the employed procedure.

- Swelling: the resin was swollen in 4 mL of DMF for 30 minutes at rt; the solvent was then ejected.
- Deprotection of the Fmoc-AA: the Fmoc-AA bound to the resin was stirred for 5 minutes at rt with 3 mL of a solution of 20% piperidine in DMF; the solution was then ejected and two more deprotections were carried in the same way. The solid was then washed using, alternatively for 2 minutes each, 3 mL of DMF and 3 mL of *iso*propanol (IPA) for four times. A last DMF washing was done.
- Coupling: the Fmoc-AA-OH to be coupled (3 eq.) was dissolved in 3 mL of DMF at rt; then, DIPEA (6 eq.) and COMU (3 eq.) were added and the resulting solution was stirred until it turned to red. This solution was added to the resin and the suspension was stirred for 1 hour at rt. The coupling solution was then ejected and the solid was washed using, alternatively for 2 minutes each, 3 mL of DMF and 3 mL of IPA for four times. A last DMF washing was done. To ensure that the coupling was complete, a few beads of resin were taken and the peptide was cleaved using 1 mL of cleavage solution (described below). The solvents were removed under reduced pressure, the peptide was dissolved in MeOH and the filtered solution was analyzed by HPLC-MS. The reaction was repeated in case a partial coupling was detected.
- Cleavage: after having coupled the remaining AAs of the peptide to be synthetized, the solid was washed three times with 3 mL DCM for 2 minutes. Then, 8 mL of a solution composed by 95% TFA, 2.5% TIS and 2.5% H_2O was added to the resin and the suspension was stirred for 1 hour at rt. Then, the solution was collected and solvents were removed under reduced pressure; TFA was azeotropically removed by two successive additions of 10 mL of PhMe and 10 mL of Et_2O were added at last. The volatiles were removed under reduced pressure. The cleavage was repeated one more time and the resin was washed with 4 mL of cleavage solution. After removing of the solvents, the crude product was obtained as a solid.

Fmoc-GPLG-OH (3)

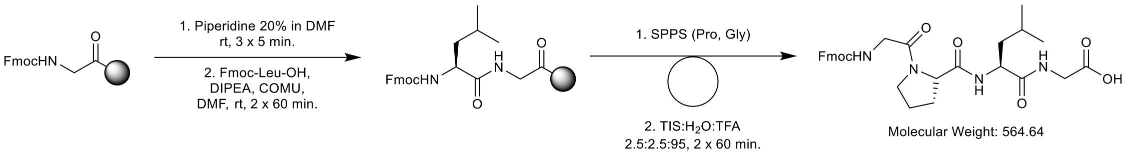

Fmoc-GPLG-OH (**3**) was obtained following the General procedure for solid phase peptide synthesis. More specifically, 0.833 g of Fmoc-Gly-Wang resin were used and the crude product was purified by flash chromatography [gradient elution from DCM/MeOH = 98:2 to DCM/MeOH = 9:1]. A white solid was obtained (0.313 g, 0.554 mmol, 99% yield).

^{1}H NMR (CD_3OD, 400 MHz, 299.2 K): δ 7.71 (d, *J* = 7.3 Hz, 2H), 7.59 (d, *J* = 6.9 Hz, 2H), 7.31 (t, *J* = 7.1 Hz, 2H), 7.24 (t, *J* = 7.1 Hz, 2H), 4.43 – 4.36 (m, 2H), 4.30 – 4.24 (m, 2H), 4.14 (t, *J* = 6.2 Hz, 1H), 3.92 (dd, *J* = 30.3, 16.8 Hz, 2H), 3.80 – 3.66 (m, 2H), 3.62 – 3.58 (m, 1H), 3.52 – 3.46 (m, 1H), 2.29 – 2.07 (m, 1H), 2.01 – 1.78 (m, 3H), 1.68 – 1.61 (m, 3H), 0.88 (d, *J* = 4.7 Hz, 3H), 0.85 (d, *J* = 4.1 Hz, 3H). ^{13}C NMR (CD_3OD, 101 MHz, 299.2 K): δ 174.73, 174.50, 171.21, 159.04, 158.91, 145.15, 145.07, 144.69, 142.43, 133.01, 132.91, 129.95, 129.82, 128.73, 128.09, 126.17, 120.87, 68.18, 62.32, 53.05, 49.85, 48.19, 47.92, 44.24, 41.02, 30.53, 25.94, 25.75, 23.59, 21.59. MS (ESI+) *m/z* calc. for $[C_{30}H_{37}N_4O_7]^+$: 565.2 $[M+H]^+$; *m/z* found: 565.9; *m/z* calc. for $[C_{60}H_{73}N_8O_{14}]^+$: 1131.4 $[2M+H]^+$; *m/z* found: 1131.2.

Fmoc-GPLGLAGG-OH

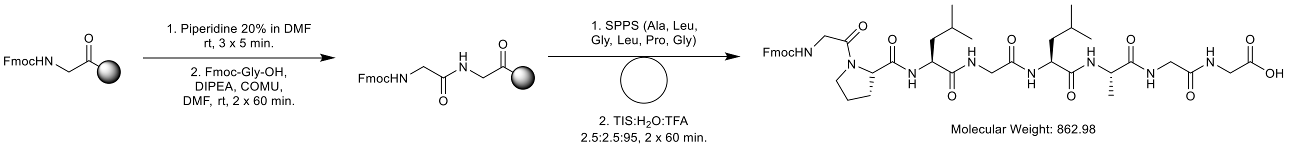

Fmoc-GPLGLAGG-OH was obtained following the General procedure for solid phase peptide synthesis. More specifically, 0.167 g of Fmoc-Gly-Wang resin were used and the crude product was purified by preparative RP-HPLC [Sepachrom Robusta 100 Å C18 5 µm 250 mm x 21.2 mm column, gradient: 95% (H_2O + 10% MeCN) / 5% (MeCN + 10% H_2O + 0.1% FA) to 100% (MeCN + 10% H_2O + 0.1% FA) in 15 min. to 100% (MeCN + 10% H_2O + 0.1% FA) in 8 min. to 95% (H_2O + 10% MeCN) / 5% (MeCN + 10% H_2O + 0.1% FA) in 7 min., t_R: 15.6 min.]. The pure fractions were concentrated under reduced pressure and the remaining aqueous solution was freeze-dried affording a white solid (0.020 g, 0.023 mmol, 23% yield).

^{1}H NMR (CD_3OD, 400 MHz, 300.0 K) δ 7.83 (d, *J* = 7.5 Hz, 2H), 7.69 (d, *J* = 7.2 Hz, 2H), 7.42 (t, *J* = 7.4 Hz, 2H), 7.34 (t, *J* = 7.3 Hz, 2H), 4.43 – 4.34 (m, 4H), 4.32 – 4.25 (m, 2H), 4.03 – 3.97 (m,

2H), 3.96 – 3.92 (m, 2H), 3.89 (s, 1H), 3.84 – 3.83 (m, 1H), 3.81 – 3.80 (m, 2H), 3.76 – 3.70 (m, 2H), 3.67 – 3.61 (m, 1H), 2.35 – 2.24 (m, 1H), 2.03 (m, 3H), 1.76 – 1.60 (m, 6H), 1.42 (m, 3H), 0.93 (m, 12H). ^{13}C NMR (CD_3OD, 101 MHz, 300.0 K) δ 175.71, 175.42, 175.38, 175.25, 175.12, 172.64, 171.92, 171.39, 159.35, 145.23, 142.61, 128.86, 128.19, 126.25, 126.22, 120.98, 68.31, 62.80, 54.04, 53.82, 51.10, 48.32, 48.00, 44.37, 44.19, 43.60, 42.03, 41.26, 40.61, 30.49, 26.10, 26.06, 25.76, 23.51, 23.45, 21.74, 21.70, 17.23. MS (ESI+) *m/z* calc. for $[C_{43}H_{59}N_8O_{11}]^+$: 863.4 $[M+H]^+$; *m/z* found: 865.0; *m/z* calc. for $[C_{86}H_{117}N_{16}O_{22}]^+$: 1725.8 $[M+H]^+$; *m/z* found: 1728.8.

Fmoc-GPLG-PAB-OH (4)

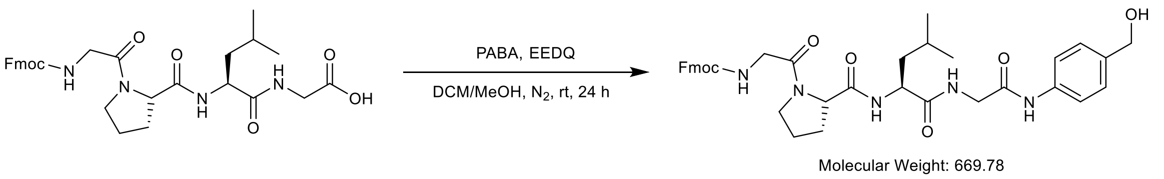

EEDQ (0.049 g, 0.20 mmol, 2 eq.) and 4-aminobenzyl alcohol (0.024 g, 0.20 mmol, 2 eq.) were added to a solution constituted by Fmoc-GPLG-OH (**3**) (0.056 g, 0.099 mmol, 1 eq.) dissolved in 1.4 mL of a dry DCM/dry MeOH = 2:1 mixture under nitrogen atmosphere. The reaction mixture was stirred in the dark for 24 hours at rt. The solvents were removed under reduced pressure, 10 mL of Et_2O were added to the crude product and the suspension was placed in a sonication bath for 10 minutes at rt. The suspension was filtered affording a white solid (0.054 g, 0.081 mmol, 82% yield).

R_f: 0.55 (DCM/MeOH=9:1). ^{1}H NMR (Acetone-d_6, 400 MHz,300.0 K) δ 8.88 (s, 1H), 7.90 (s, 1H), 7.85 (d, J = 7.5 Hz, 2H), 7.78 (d, J = 8.2 Hz, 2H), 7.69 (d, J = 5.3 Hz, 2H), 7.40 (t, J = 7.4 Hz, 2H), 7.32 (t, J = 7.4 Hz, 2H), 7.26 (d, J = 8.3 Hz, 2H), 6.73 (s, 1H), 4.54 (s, 2H), 4.42 (dd, J = 8.1, 4.4 Hz, 1H), 4.31 (d, J = 7.1 Hz, 2H), 4.28 – 4.19 (m, 2H), 4.04 (s, 1H), 3.92 (d, J = 9.8 Hz, 4H), 3.69 (dd, J = 14.1, 7.0 Hz, 2H), 2.80 (d, J = 12.5 Hz, 2H), 2.31 – 2.19 (m, 1H), 2.13 – 2.07 (m, 1H), 1.76 – 1.63 (m, 3H), 0.91 (d, J = 5.6 Hz, 3H), 0.89 (d, J = 5.6 Hz, 3H). ^{13}C NMR (Acetone-d_6, 101 MHz, 300.1 K) δ 174.02 (conformer 1), 173.94 (conformer 2), 173.00 (conformer 1), 172.90 (conformer 2), 170.38 (conformer 1), 168.53 (conformer 1), 168.46 (conformer 2), 157.78 (conformer 1), 145.14 (conformer 1), 144.93 (conformer 2), 142.13 (conformer 2), 142.10 (conformer 1), 138.83 (conformer 1), 138.74 (conformer 2), 138.55 (conformer 1), 128.57 (conformer 2), 128.55 (conformer 1), 127.98 (conformer 1), 127.88 (conformer 2), 127.79 (conformer 1), 126.19 (conformer 2), 126.13 (conformer 1), 120.82 (conformer 1), 120.09 (conformer 1), 120.01 (conformer 2), 67.52 (conformer 1), 64.45 (conformer 1), 64.33 (conformer 2), 62.42 (conformer 1), 62.39 (conformer 2), 53.83 (conformer 1), 53.73 (conformer 2), 47.92 (conformer 1), 47.58 (conformer 1), 44.23 (conformer 1), 44.13

(conformer 1), 40.04 (conformer 1), 39.99 (conformer 2), 29.94 (conformer 1), 25.79 (conformer 1), 25.71 (conformer 1), 23.49 (conformer 1), 21.65 (conformer 1).

MS (ESI+) *m/z* calc. for $[C_{37}H_{44}N_5O_7]^+$: 670.3 $[M+H]^+$; *m/z* found: 671.3; *m/z* calc. for $[C_{74}H_{87}N_{10}O_{14}]^+$: 1339.6 $[2M+H]^+$; *m/z* found: 1341.8.

Fmoc-GPLG-*N*-benzyl (4-nitrophenyl) carbonate (5)

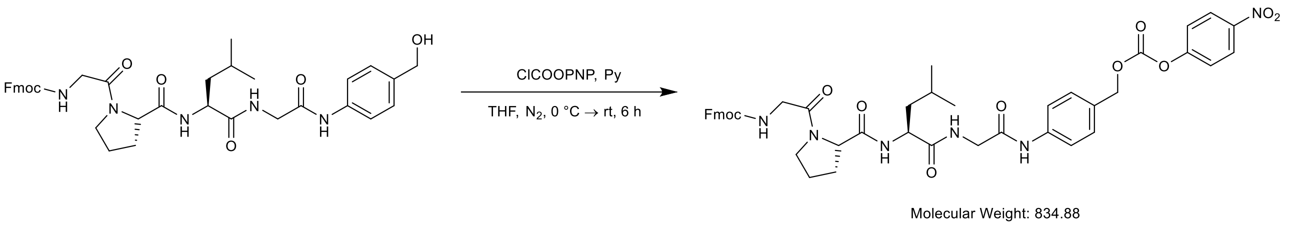

Pyridine (0.038 mL, 0.47 mmol, 3 eq.) and 4-nitrophenyl chloroformate (0.063 g, 0.31 mmol, 2 eq.) were added to a 0 °C solution of Fmoc-GPLG-PAB-OH (**4**) (0.105 g, 0.16 mmol, 1 eq.) dissolved in 2.6 mL of dry THF under nitrogen atmosphere. The reaction mixture was stirred for 1.5 hours at rt. Then, additional pyridine (0.025 mL, 0.31 mmol, 2 eq.) and 4-nitrophenyl chloroformate (0.032 g, 0.16 mmol, 1 eq.) were added to the reaction mixture and it was stirred for 2.5 hours at rt. An extra amount of 4-nitrophenyl chloroformate (0.032 g, 0.16 mmol, 1 eq.) was added to the reaction mixture and it was stirred for 2 hours at rt. The suspension was filtered and the solvent was removed under reduced pressure. The crude product was purified by flash chromatography [eluent: DCM/acetone = 6.4:3.6] affording a white solid (0.061 g, 0.073 mmol, 47% yield).

R_f: 0.61 (DCM/acetone=6.4:3.6). MS (ESI+) *m/z* calc. for $[C_{44}H_{47}N_6O_{11}]^+$: 835.3 $[M+H]^+$; *m/z* found: 836.6; *m/z* calc. for $[C_{88}H_{93}N_{12}O_{22}]^+$: 1669.6 $[2M+H]^+$; *m/z* found: 1672.0.

Fmoc-GPLG-PABC-MMAE (6)

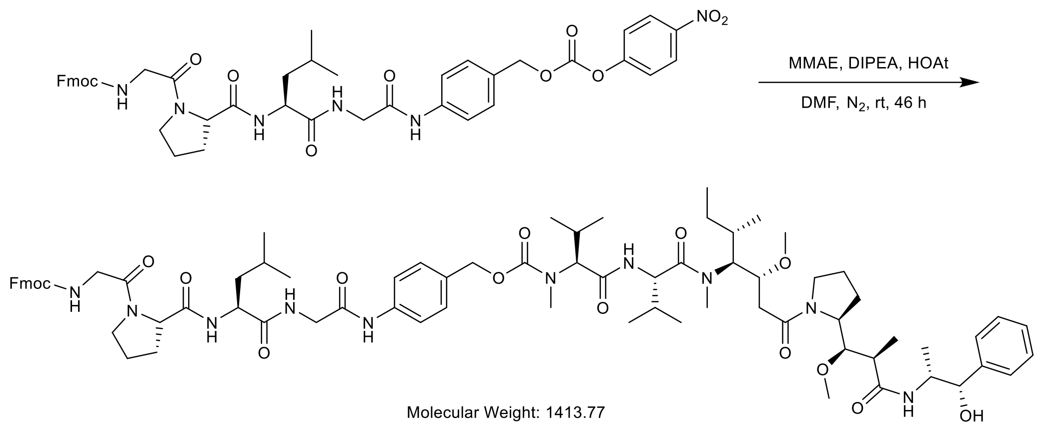

MMAE (0.008 g, 0.01 mmol, 1 eq.) and DIPEA (0.013 mL, 0.07 mmol, 6 eq.) were added to a solution constituted by Fmoc-GPLG-*N*-benzyl (4-nitrophenyl) carbonate (**5**) (0.019 g, 0.02 mmol, 2 eq.) dissolved in 0.22 mL of dry DMF under nitrogen atmosphere. The reaction mixture was stirred for 19 hours at rt. Then, a 0.6 mol/L solution of HOAt (0.009 mL, 0.006 mmol, 0.5 eq.) in DMF were added followed by other Fmoc-GPLG-*N*-benzyl (4-nitrophenyl) carbonate (**5**) (0.019 g, 0.02 mmol, 2 eq.) and the reaction mixture was stirred for 7 hours at rt. A last amount of Fmoc-GPLG-*N*-benzyl (4-nitrophenyl) carbonate (**5**) (0.010 g, 0.01 mmol, 1 eq.) was added to the reaction mixture and it was stirred for 20 hours at rt. The reaction mixture was concentrated under reduced pressure and the crude residue was purified by preparative RP-HPLC [Sepachrom Robusta 100 Å C18 5 µm 250 mm x 21.2 mm column, gradient: 95% (H_2O + 10% MeCN) / 5% (MeCN + 10% H_2O + 0.1% FA) to 100% (MeCN + 10% H_2O + 0.1% FA) in 15 min. to 100% (MeCN + 10% H_2O + 0.1% FA) in 8 min. to 95% (H_2O + 10% MeCN) / 5% (MeCN + 10% H_2O + 0.1% FA) in 7 min., t_R: 19.9 min.]. The pure fractions were concentrated under reduced pressure and the remaining aqueous solution was freeze-dried affording a white solid (0.012 g, 0.008 mmol, 73% yield).

MS (ESI+) *m/z* calc. for $[C_{77}H_{109}N_{10}O_{15}]^+$: 1413.8 $[M+H]^+$; *m/z* found: 1416.4.

H_2N-GPLG-PABC-MMAE (7)

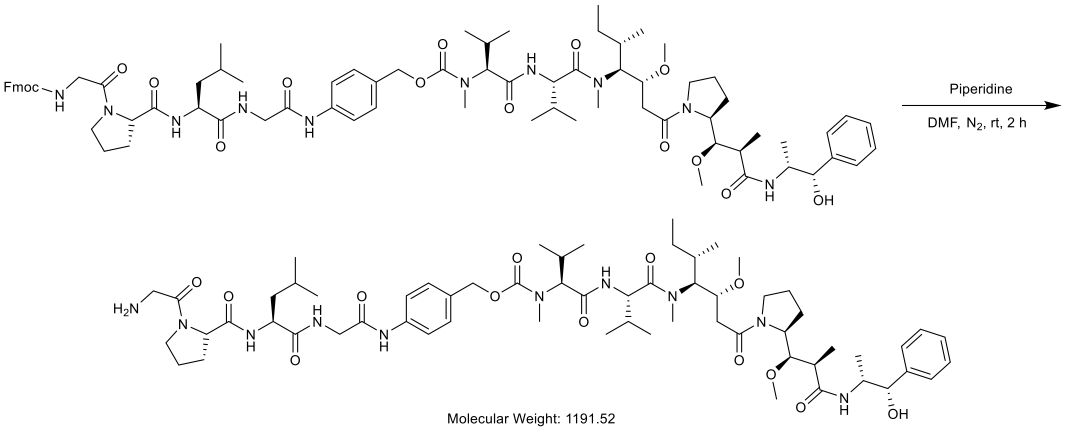

Piperidine (0.044 mL, 20% of the total volume) was added to a solution constituted by Fmoc-GPLG-PABC-MMAE (**6**) (0.014 g, 0.001 mmol, 1 eq.) dissolved in 0.174 mL of dry DMF under nitrogen atmosphere. The reaction mixture was stirred for 2 hours at rt. Then, it was concentrated under reduced pressure and the crude residue was purified by preparative HPLC [Sepachrom Robusta 100 Å C18 5 µm 250 mm x 21.2 mm column, gradient: 95% (H_2O + 10% MeCN) / 5% (MeCN + 10% H_2O + 0.1% FA) to 40% (H_2O + 10% MeCN) / 60% (MeCN + 10% H_2O + 0.1% FA) in 18 min. to 10% (H_2O + 10% MeCN) / 90% (MeCN + 10% H_2O + 0.1% FA) in 7 min. to 10% (H_2O + 10% MeCN) / 90% (MeCN + 10% H_2O + 0.1% FA) in 5 min. to 90% (H_2O + 10% MeCN) / 10% (MeCN + 10% H_2O + 0.1% FA) in 5 min. to 90% (H_2O + 10% MeCN) / 10% (MeCN + 10% H_2O + 0.1% FA) in 1 min., t_R: 16 min.]. The pure fractions were concentrated under reduced pressure and the remaining aqueous solution was freeze-dried affording a white solid (0.007 g, 0.006 mmol, 61% yield).

MS (ESI+) *m/z* calc. for $[C_{62}H_{99}N_{10}O_{13}]^+$: 1191.7 $[M+H]^+$; *m/z* found: 1193.7.

HRMS (ESI+): *m/z* calc. for $[C_{62}H_{99}N_{10}O_{13}]^+$: 1191.7393 $[M+2H]^{2+}$; *m/z* found: 1191.7397.

4-pentynoic amide-*N*-(GPLG-PABC-MMAE) (8)

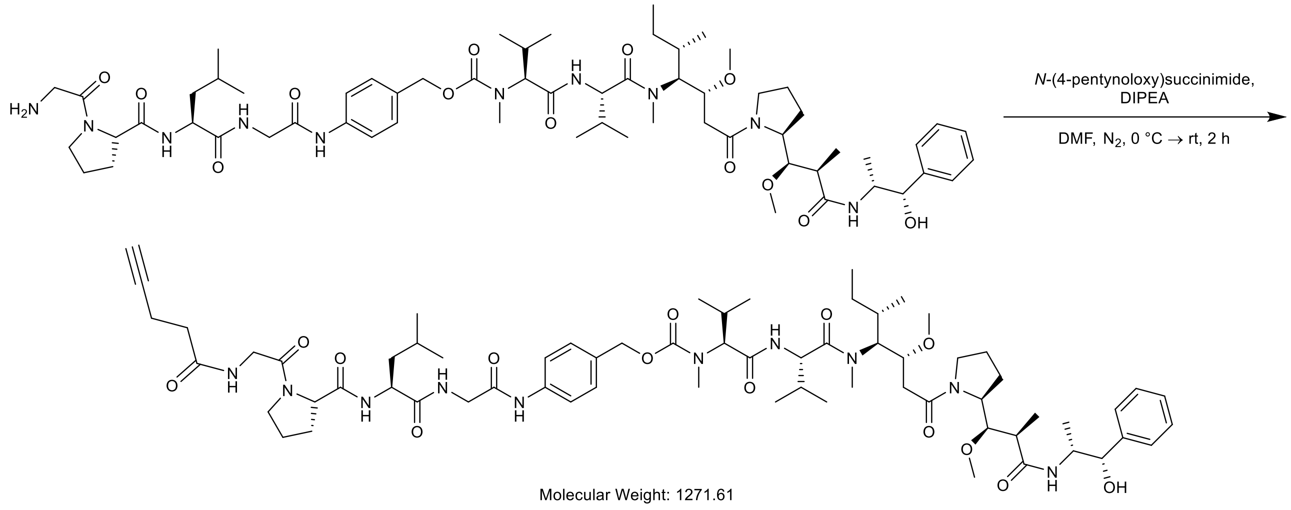

N-(4-pentynoloxy)succinimide[1] (0.0034 g, 0.017 mmol, 2.5 eq.) and DIPEA (0.005 mL, 0.029 mmol, 4 eq.) were added to a solution, cooled at 0 °C, constituted by H_2N-GPLG-PABC-MMAE (**7**) (0.0083 g, 0.007 mmol, 1 eq.) dissolved in 0.15 mL of dry DMF under nitrogen atmosphere. The reaction mixture was stirred for 2 hours at rt. Then, it was concentrated under reduced pressure and the crude residue was purified by preparative HPLC [Sepachrom Robusta 100 Å C18 5 µm 250 mm x 21.2 mm column, gradient: 95% (H_2O + 10% MeCN) / 5% (MeCN + 10% H_2O + 0.1% FA) to 100% (MeCN + 10% H_2O + 0.1% FA) in 15 min. to 100% (MeCN + 10% H_2O + 0.1% FA) in 8 min. to 95% (H_2O + 10% MeCN) / 5% (MeCN + 10% H_2O + 0.1% FA) in 7 min., t_R: 15.6 min.]. The pure fractions were concentrated under reduced pressure and the remaining aqueous solution was freeze-dried affording a white solid (0.0032 g, 0.003 mmol, 36% yield).

MS (ESI+) *m/z* calc. for $[C_{67}H_{103}N_{10}O_{14}]^+$: 1271.8 $[M+H]^+$; *m/z* found: 1274.0.

cyclo[DKP-*iso*DGR]-PEG-4-GPLG-PABC-MMAE (2)

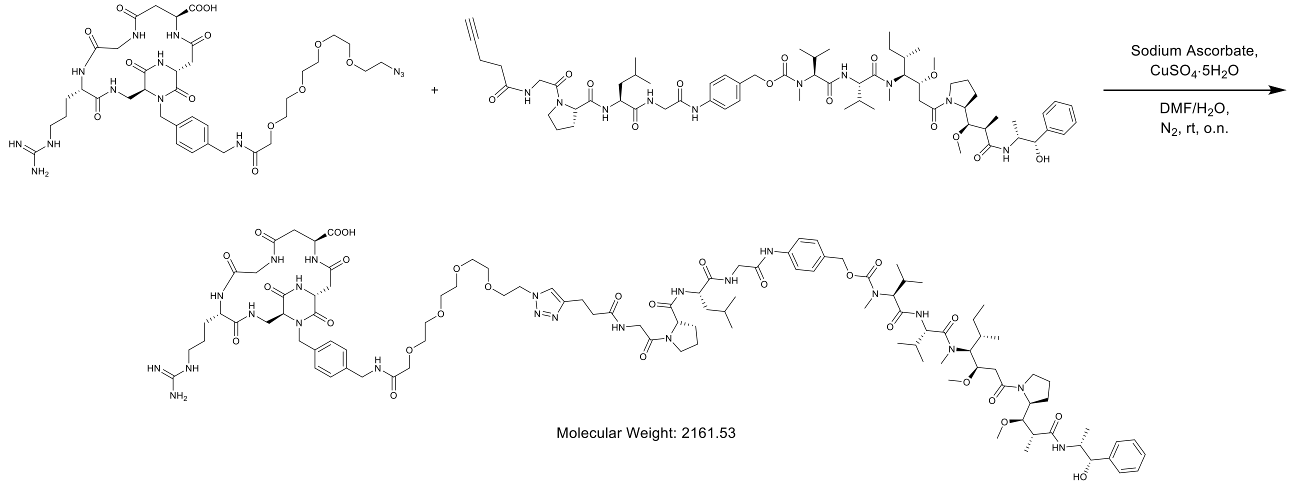

A solution of *cyclo*[DKP-*iso*DGR]-PEG-4-N_3[1] (**9**) (0.0034 g, 0.0038 mmol, 1.5 eq.) dissolved in 0.15 mL of degassed water under nitrogen atmosphere was added to a solution constituted by 4-pentynoic amide-*N*-(GPLG-PABC-MMAE) (**8**) (0.0032 g, 0.0025 mmol, 1 eq.) dissolved in 0.2 mL of dry DMF under nitrogen atmosphere. Then, a 3 g/L solution of sodium ascorbate (0.066 mL, 0.001 mmol, 0.4 eq.) in degassed water and a 3 g/L solution of $CuSO_4 \cdot 5H_2O$ (0.042 mL, 0.0005 mmol, 0.2 eq.) in degassed water were added; the reaction mixture was stirred for 7 hours at rt. An additional amount of the 3 g/L solution of sodium ascorbate (0.033 mL, 0.0005 mmol, 0.2 eq.) in degassed water and of the 3 g/L solution of $CuSO_4 \cdot 5H_2O$ (0.021 mL, 0.0003 mmol, 0.1 eq.) in degassed water were added and the reaction mixture was stirred for 18 hours at rt. Then, it was concentrated under reduced pressure and the crude residue was purified by preparative HPLC [Sepachrom Robusta 100 Å C18 5 µm 250 mm x 21.2 mm column, gradient: 95% (H_2O + 10% MeCN) / 5% (MeCN + 10% H_2O + 0.1% FA) to 40% (H_2O + 10% MeCN) / 60% (MeCN + 10% H_2O + 0.1% FA) in 18 min. to 10% (H_2O + 10% MeCN) / 90% (MeCN + 10% H_2O + 0.1% FA) in 7 min. to 10% (H_2O + 10% MeCN) / 90% (MeCN + 10% H_2O + 0.1% FA) in 5 min. to 90% (H_2O + 10% MeCN) / 10% (MeCN + 10% H_2O + 0.1% FA) in 5 min. to 90% (H_2O + 10% MeCN) / 10% (MeCN + 10% H_2O + 0.1% FA) in 1 min., t_R: 16 min.]. The pure fractions were concentrated under reduced pressure and the remaining aqueous solution was freeze-dried affording a white solid (0.0016 g, 0.0007 mmol, 30% yield).

MS (ESI+) *m/z* calc. for $[C_{104}H_{159}N_{23}O_{27}]^{2+}$: 1081.1 $[M+2H]^{2+}$; *m/z* found: 1082.7. HRMS (ESI-): *m/z* calc. for $[C_{104}H_{156}N_{23}O_{27}]^{-}$: 2159.1546 $[M-1H]^{-}$; *m/z* found: 2159.1546; *m/z* calc. for $[C_{104}H_{155}N_{23}O_{27}]^{2-}$: 1079.0737 $[M-2H]^{2-}$; *m/z* found: 1079.0736. HRMS (ESI+): *m/z* calc. for $[C_{104}H_{159}N_{23}O_{27}]^{2+}$: 1081.0882 $[M+2H]^{2+}$; *m/z* found: 1081.0868; *m/z* calc. for $[C_{104}H_{160}N_{23}O_{27}]^{3+}$: 721.0613 $[M+3H]^{3+}$; *m/z* found: 721.0607.

S2. TIC and MS spectra traces

Fmoc-GPLG-OH (3)

Phenomenex 150 mm x 4.6 mm Synergi 4 µm Fusion-RP 80 Å column, gradient: 90% (H_2O + 5% MeCN + 0.1% FA) / 10% (MeCN + 5% H_2O + 0.1% FA) to 10% (H_2O + 5% MeCN + 0.1% FA) / 90% (MeCN + 5% H_2O + 0.1% FA) in 8 min. to 5% (H_2O + 5% MeCN + 0.1% FA) / 95% (MeCN + 5% H_2O + 0.1% FA) in 7 min. to 5% (H_2O + 5% MeCN + 0.1% FA) / 95% (MeCN + 5% H_2O + 0.1% FA) in 5 min.

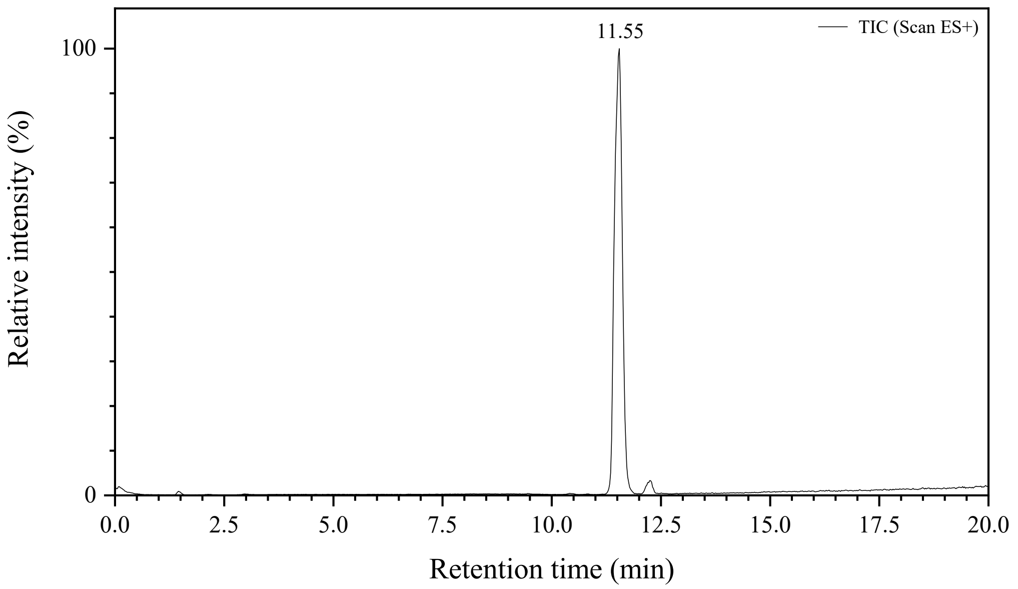

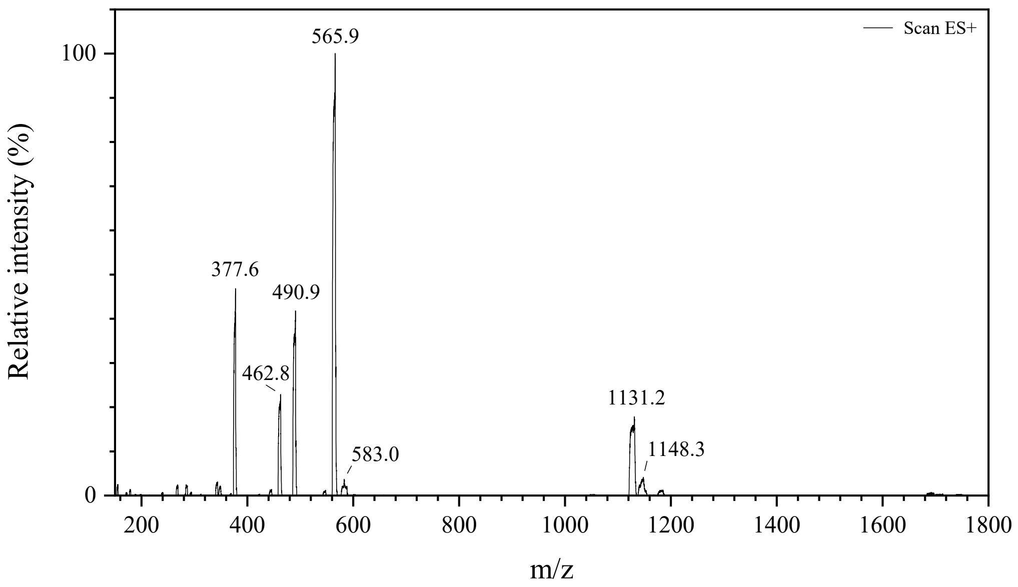

Fmoc-GPLGLAGG-OH

Phenomenex 150 mm x 4.6 mm Synergi 4 µm Fusion-RP 80 Å column, gradient: 90% (H_2O + 5% MeCN + 0.1% FA) / 10% (MeCN + 5% H_2O + 0.1% FA) to 10% (H_2O + 5% MeCN + 0.1% FA) / 90% (MeCN + 5% H_2O + 0.1% FA) in 8 min. to 5% (H_2O + 5% MeCN + 0.1% FA) / 95% (MeCN + 5% H_2O + 0.1% FA) in 7 min. to 5% (H_2O + 5% MeCN + 0.1% FA) / 95% (MeCN + 5% H_2O + 0.1% FA) in 5 min.

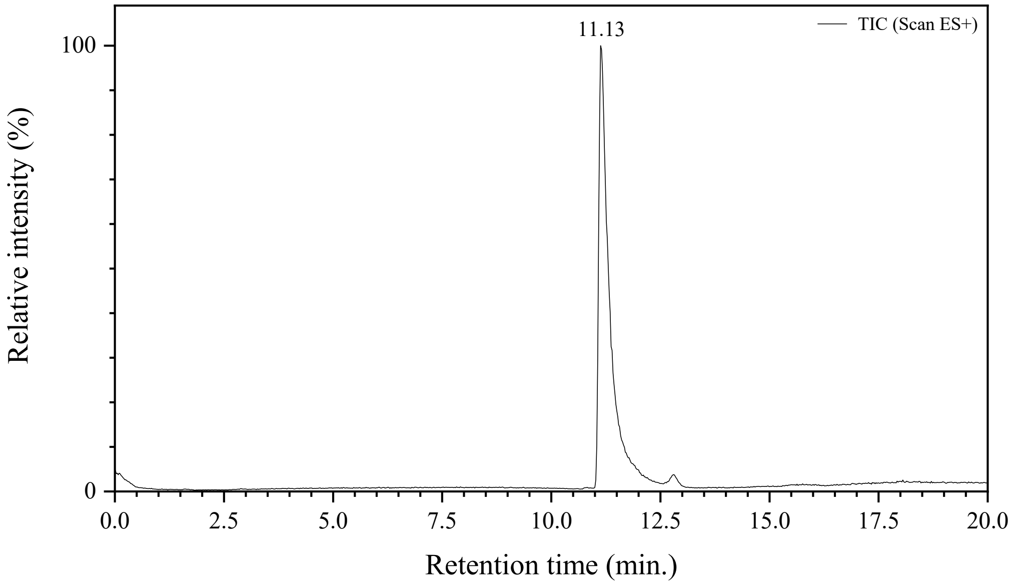

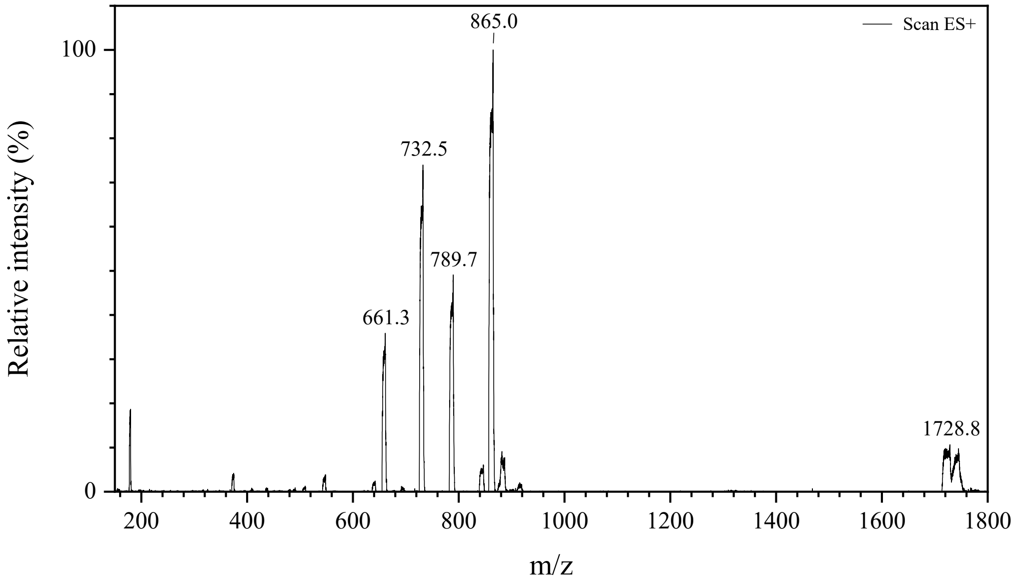

Fmoc-GPLG-PAB-OH (4)

Phenomenex 150 mm x 4.6 mm Synergi 4 µm Fusion-RP 80 Å column, gradient: 90% (H_2O + 5% MeCN + 0.1% FA) / 10% (MeCN + 5% H_2O + 0.1% FA) to 10% (H_2O + 5% MeCN + 0.1% FA) / 90% (MeCN + 5% H_2O + 0.1% FA) in 8 min. to 5% (H_2O + 5% MeCN + 0.1% FA) / 95% (MeCN + 5% H_2O + 0.1% FA) in 7 min. to 5% (H_2O + 5% MeCN + 0.1% FA) / 95% (MeCN + 5% H_2O + 0.1% FA) in 5 min.

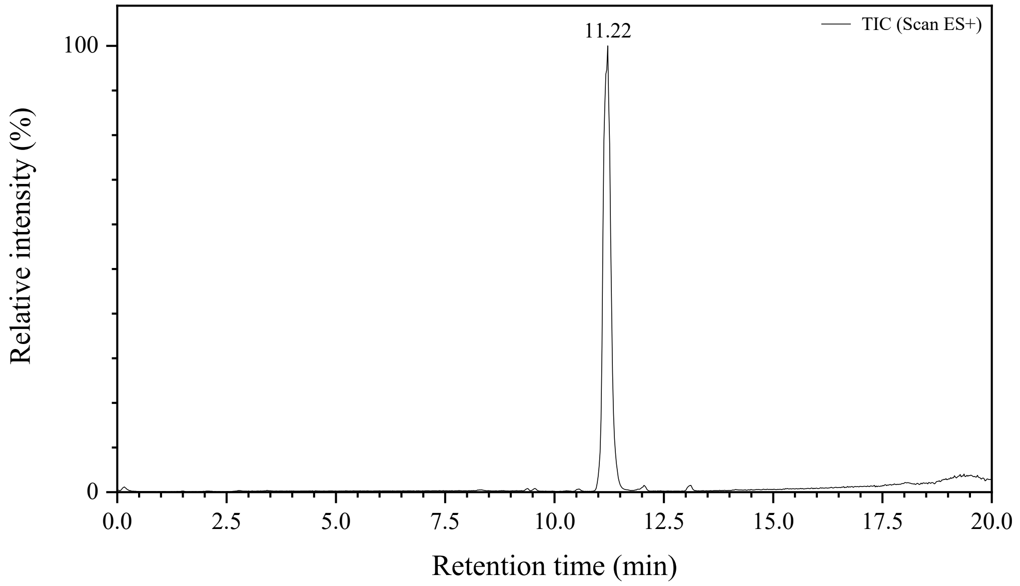

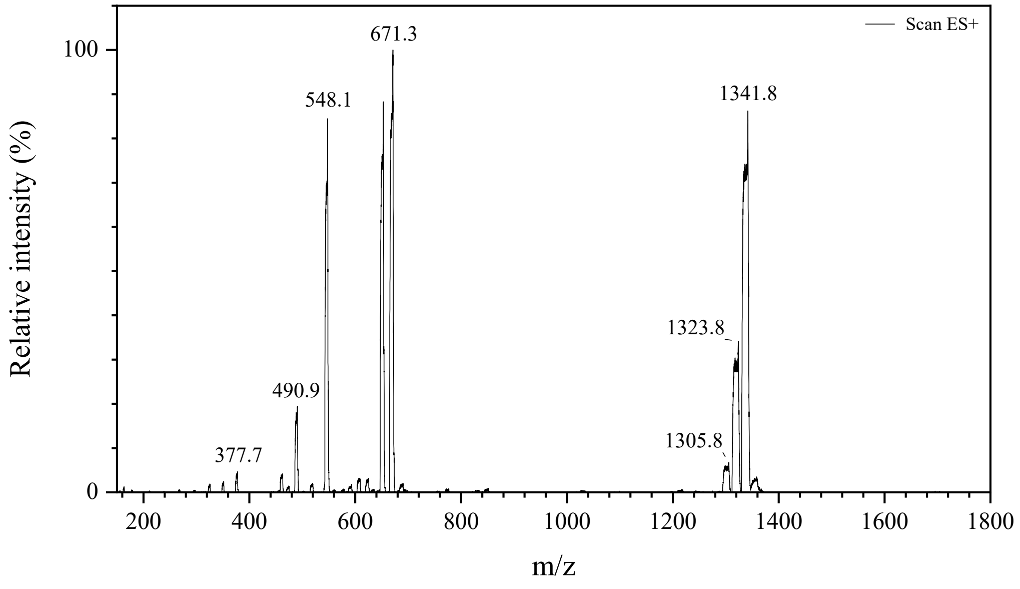

Fmoc-GPLG-*N*-benzyl (4-nitrophenyl) carbonate (5)

Phenomenex 150 mm x 4.6 mm Synergi 4 µm Fusion-RP 80 Å column, gradient: 90% (H_2O + 5% MeCN + 0.1% FA) / 10% (MeCN + 5% H_2O + 0.1% FA) to 10% (H_2O + 5% MeCN + 0.1% FA) / 90% (MeCN + 5% H_2O + 0.1% FA) in 8 min. to 5% (H_2O + 5% MeCN + 0.1% FA) / 95% (MeCN + 5% H_2O + 0.1% FA) in 7 min. to 5% (H_2O + 5% MeCN + 0.1% FA) / 95% (MeCN + 5% H_2O + 0.1% FA) in 5 min.

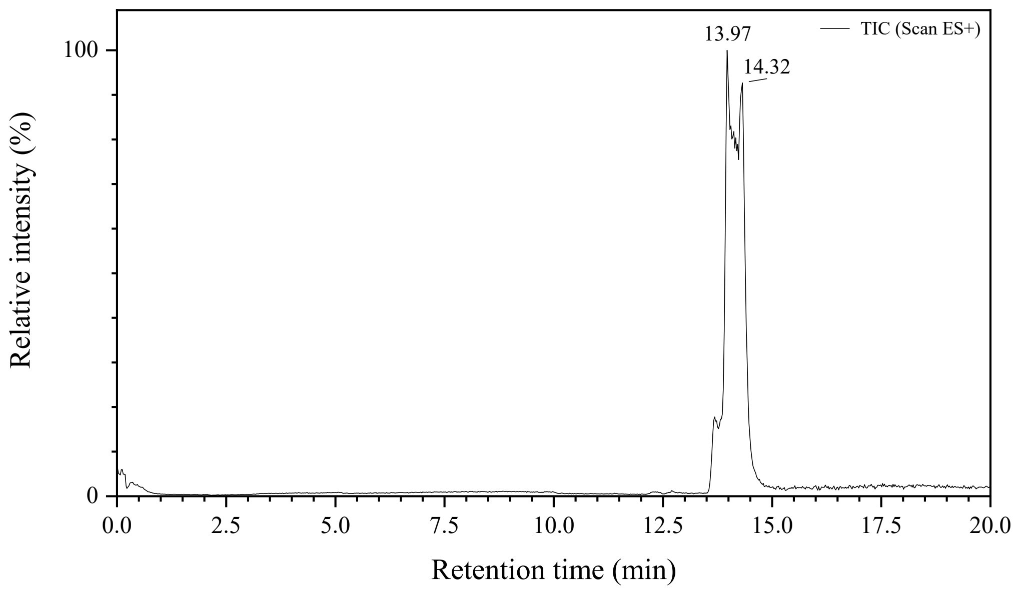

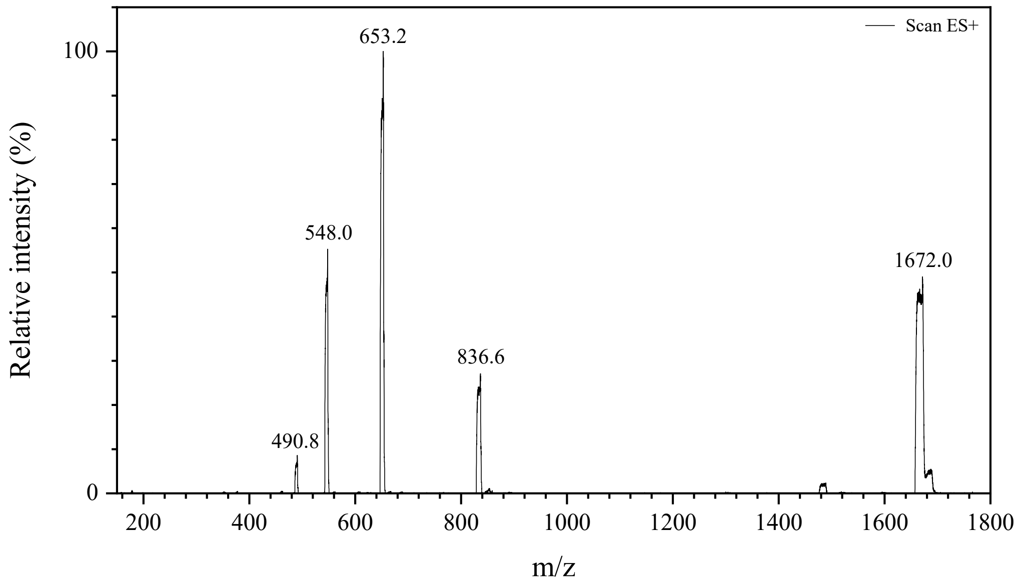

Fmoc-GPLG-PABC-MMAE (6)

Phenomenex 150 mm x 4.6 mm Synergi 4 µm Fusion-RP 80 Å column, gradient: 90% (H_2O + 5% MeCN + 0.1% FA) / 10% (MeCN + 5% H_2O + 0.1% FA) to 10% (H_2O + 5% MeCN + 0.1% FA) / 90% (MeCN + 5% H_2O + 0.1% FA) in 8 min. to 5% (H_2O + 5% MeCN + 0.1% FA) / 95% (MeCN + 5% H_2O + 0.1% FA) in 7 min. to 5% (H_2O + 5% MeCN + 0.1% FA) / 95% (MeCN + 5% H_2O + 0.1% FA) in 5 min.

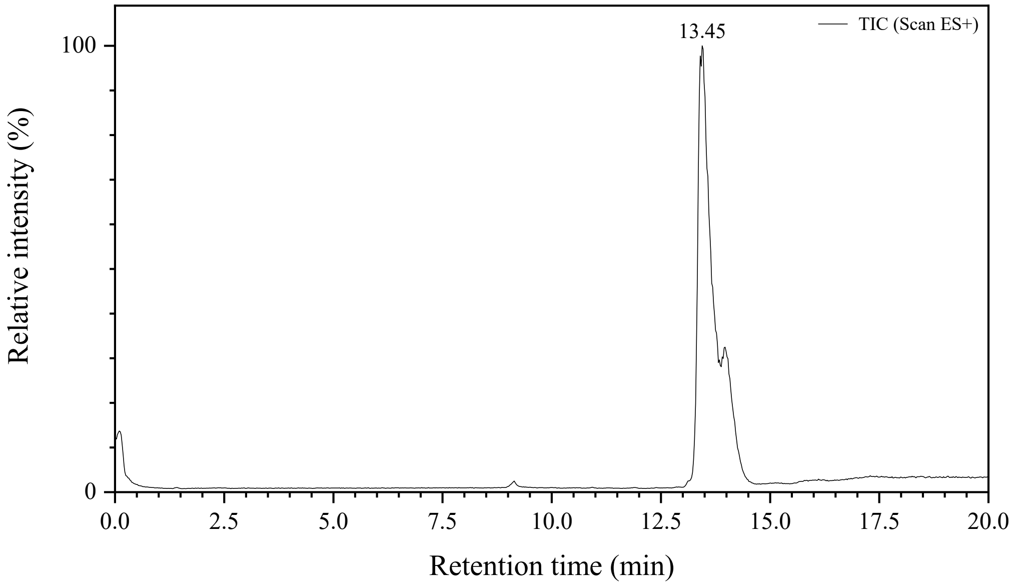

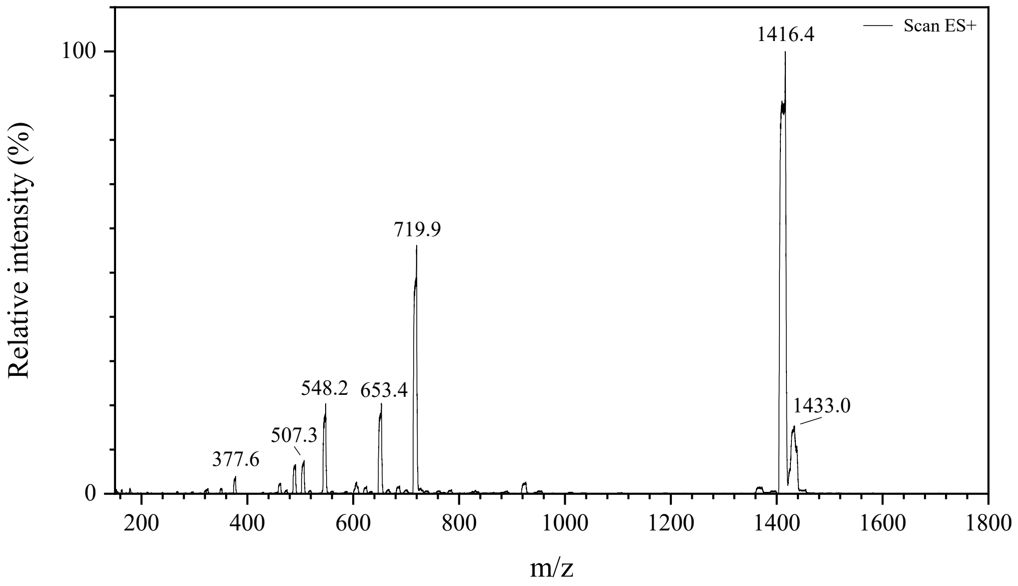

H_2N-GPLG-PABC-MMAE (7)

Phenomenex 150 mm x 4.6 mm Synergi 4 µm Fusion-RP 80 Å column, gradient: 90% (H_2O + 5% MeCN + 0.1% FA) / 10% (MeCN + 5% H_2O + 0.1% FA) to 10% (H_2O + 5% MeCN + 0.1% FA) / 90% (MeCN + 5% H_2O + 0.1% FA) in 8 min. to 5% (H_2O + 5% MeCN + 0.1% FA) / 95% (MeCN + 5% H_2O + 0.1% FA) in 7 min. to 5% (H_2O + 5% MeCN + 0.1% FA) / 95% (MeCN + 5% H_2O + 0.1% FA) in 5 min.

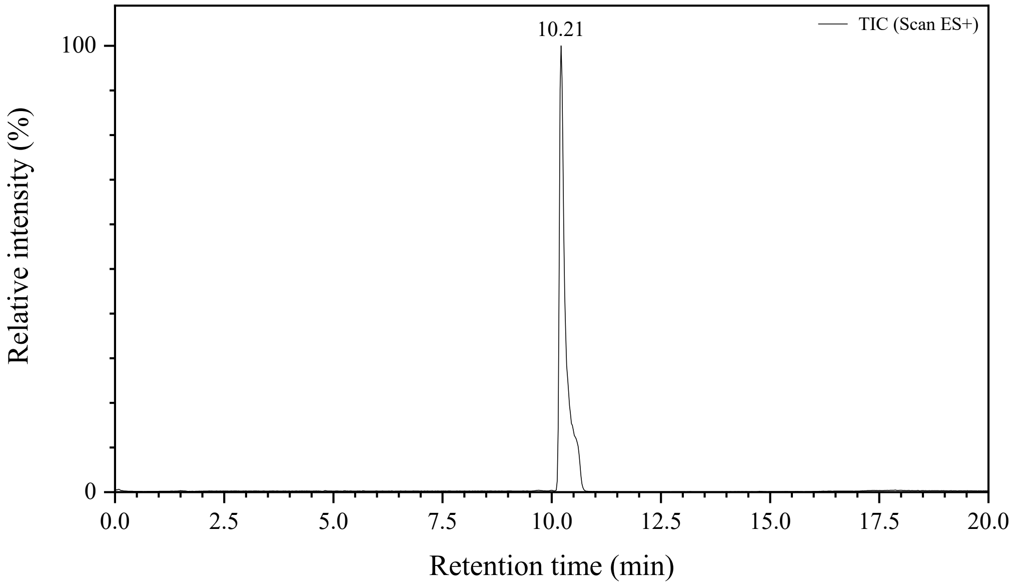

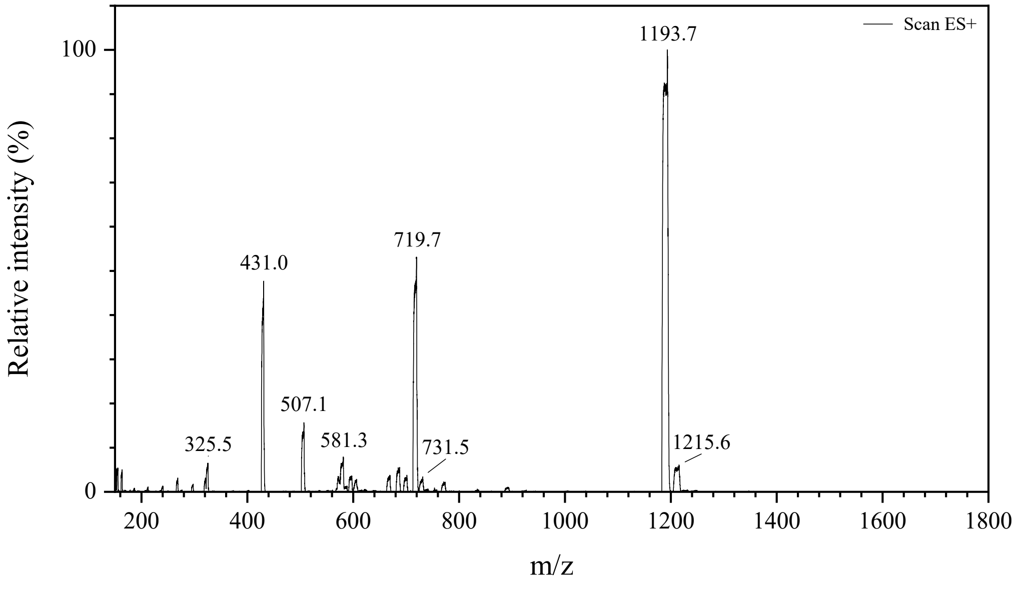

4-pentynoic-amide-*N*-(GPLG-PABC-MMAE) (8)

Phenomenex 150 mm x 4.6 mm Synergi 4 µm Fusion-RP 80 Å column, gradient: 90% (H_2O + 5% MeCN + 0.1% FA) / 10% (MeCN + 5% H_2O + 0.1% FA) to 10% (H_2O + 5% MeCN + 0.1% FA) / 90% (MeCN + 5% H_2O + 0.1% FA) in 8 min. to 5% (H_2O + 5% MeCN + 0.1% FA) / 95% (MeCN + 5% H_2O + 0.1% FA) in 7 min. to 5% (H_2O + 5% MeCN + 0.1% FA) / 95% (MeCN + 5% H_2O + 0.1% FA) in 5 min.

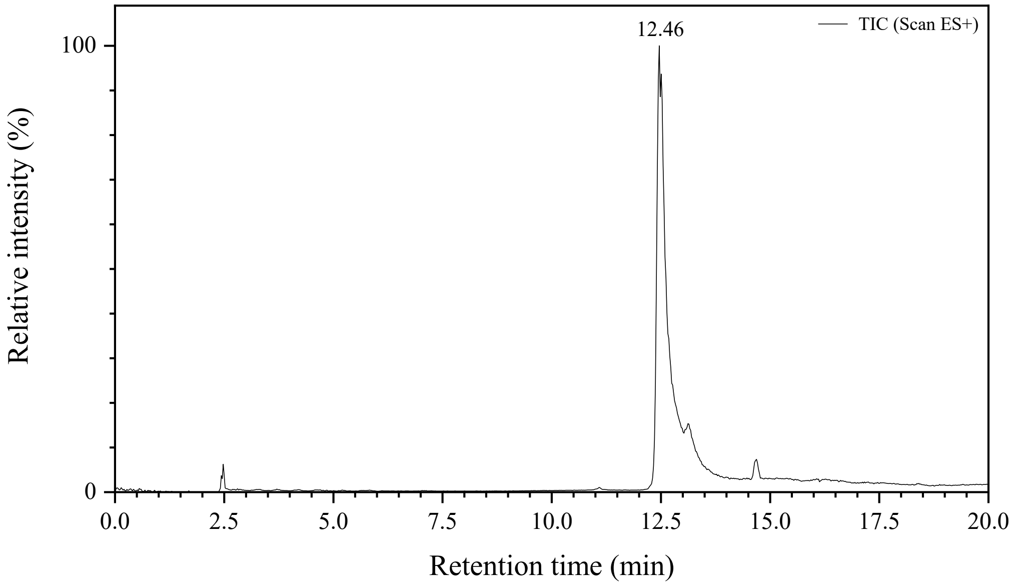

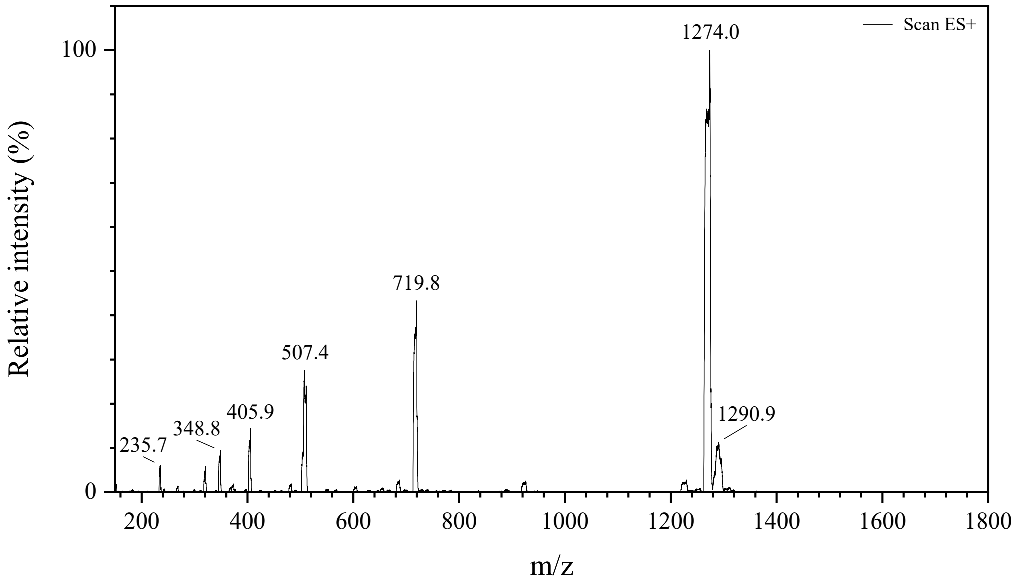

***cyclo*[DKP-*iso*DGR]-PEG-4-GPLG-PABC-MMAE (2)**

Phenomenex 150 mm x 4.6 mm Synergi 4 µm Fusion-RP 80 Å column, gradient: 90% (H_2O + 5% MeCN + 0.1% FA) / 10% (MeCN + 5% H_2O + 0.1% FA) to 10% (H_2O + 5% MeCN + 0.1% FA) / 90% (MeCN + 5% H_2O + 0.1% FA) in 8 min. to 5% (H_2O + 5% MeCN + 0.1% FA) / 95% (MeCN + 5% H_2O + 0.1% FA) in 7 min. to 5% (H_2O + 5% MeCN + 0.1% FA) / 95% (MeCN + 5% H_2O + 0.1% FA) in 5 min.

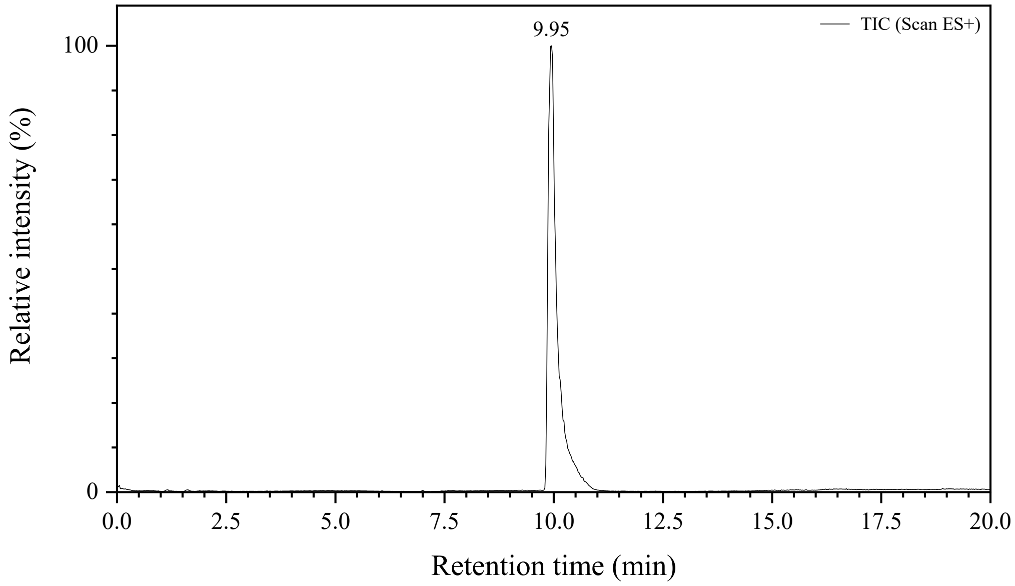

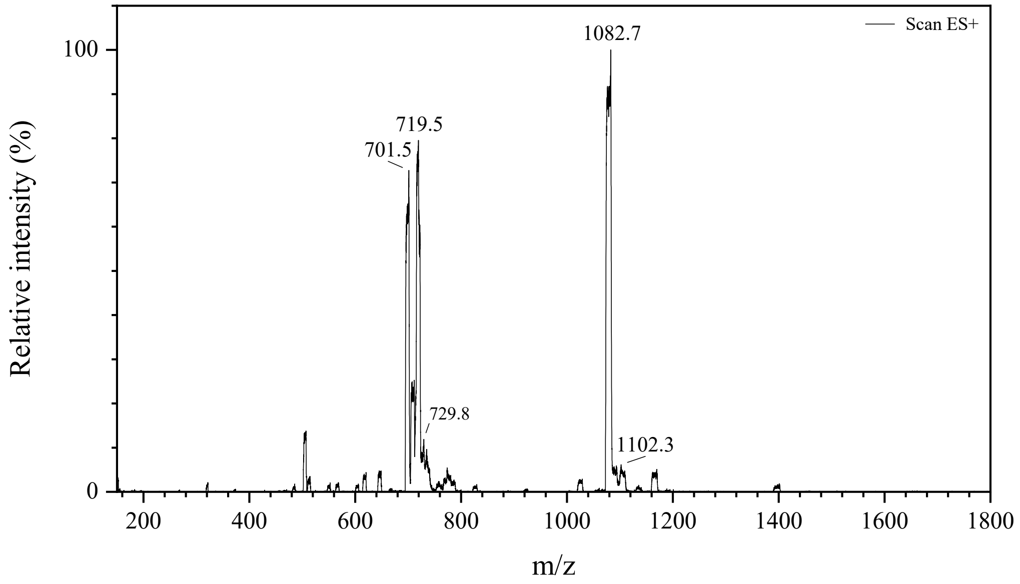

S3. ^{1}H-NMR and ^{13}C-NMR spectra

Figure S3.1 ^{1}H-NMR spectrum (CD_3OD, 400 MHz) of Fmoc-GPLGLAGG-OH

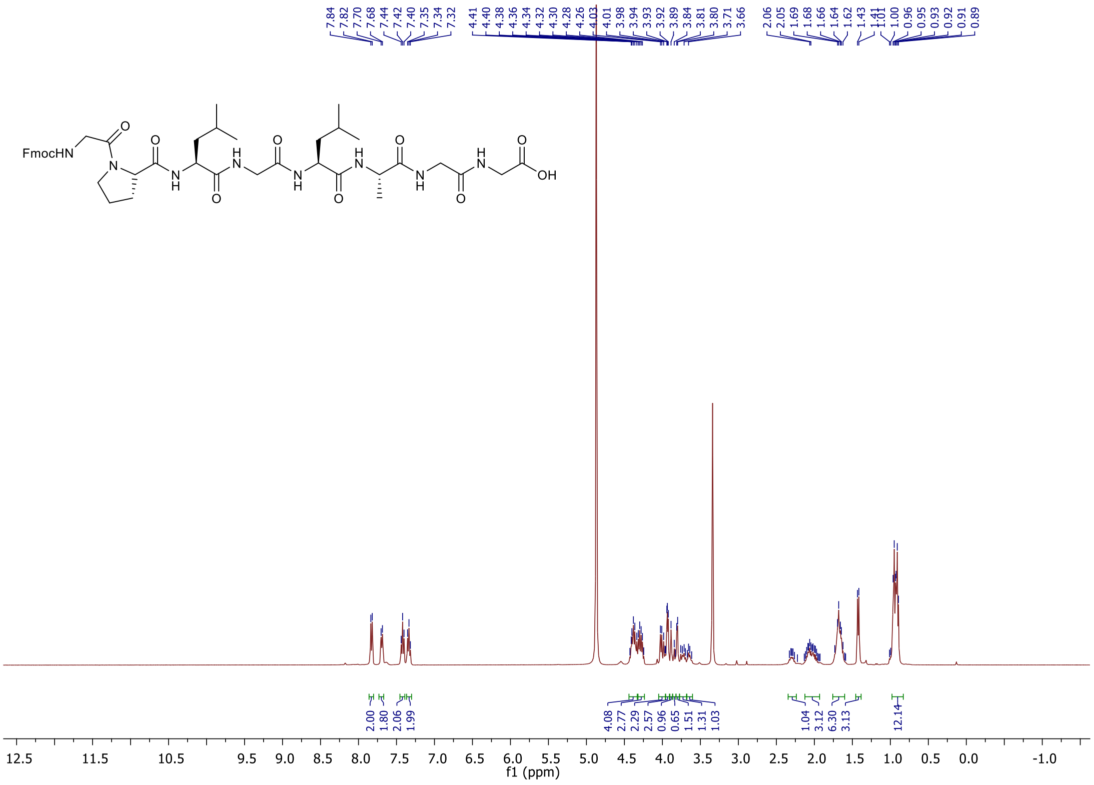

Figure S3.2 ^{13}C-JMOD NMR spectrum (CD_3OD, 101 MHz) of Fmoc-GPLGLAGG-OH

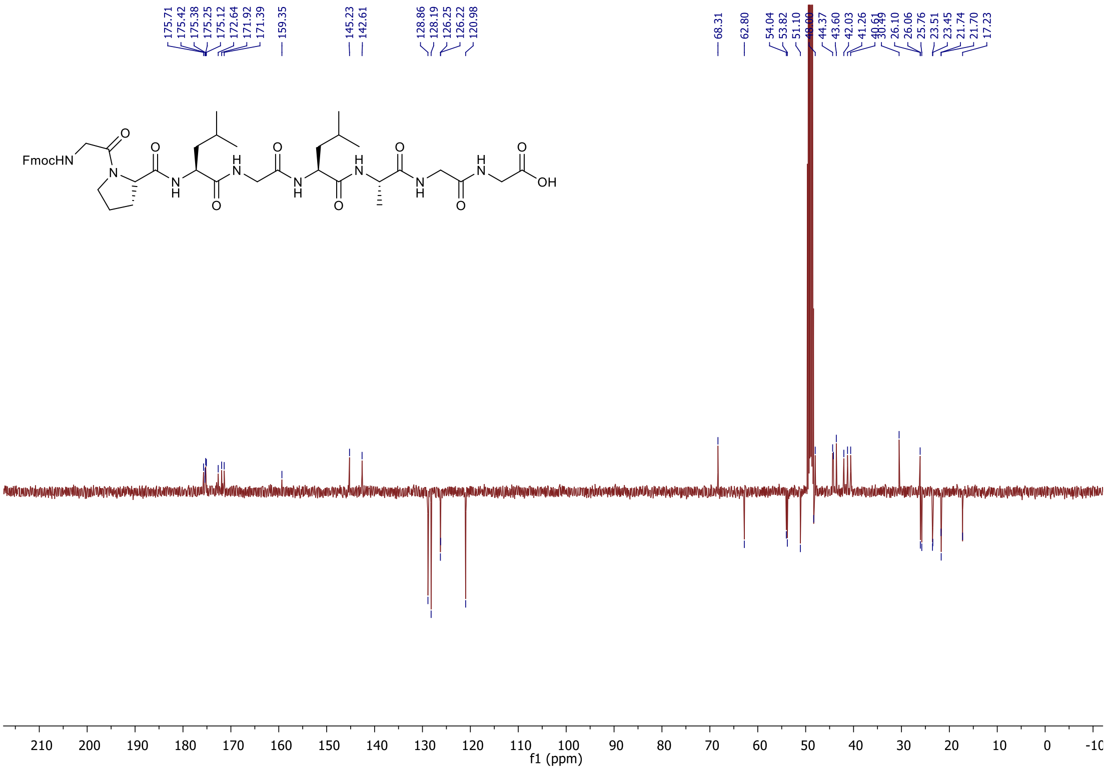

Figure S3.3 ^{1}H-NMR spectrum (CD_3OD, 400 MHz) of Fmoc-GPLG-OH (3)

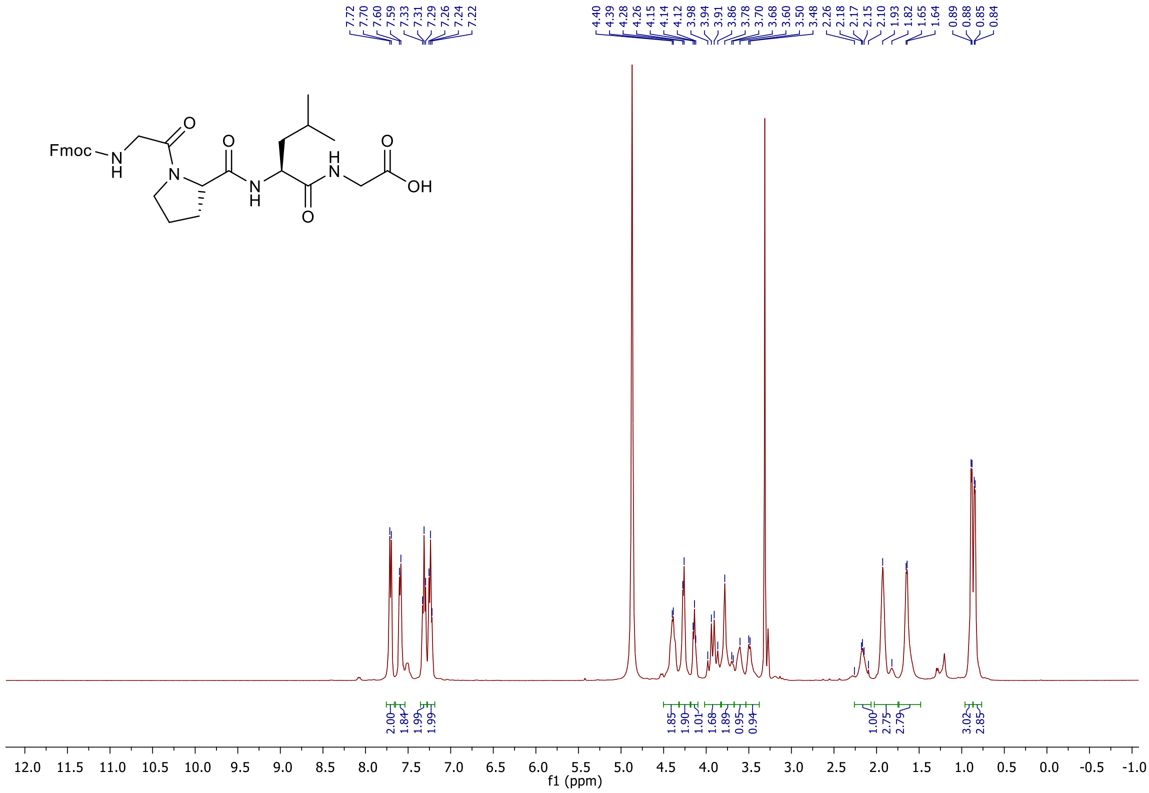

Figure S3.4 ^{13}C-JMOD NMR spectrum (CD_3OD, 101 MHz) of Fmoc-GPLG-OH (3)

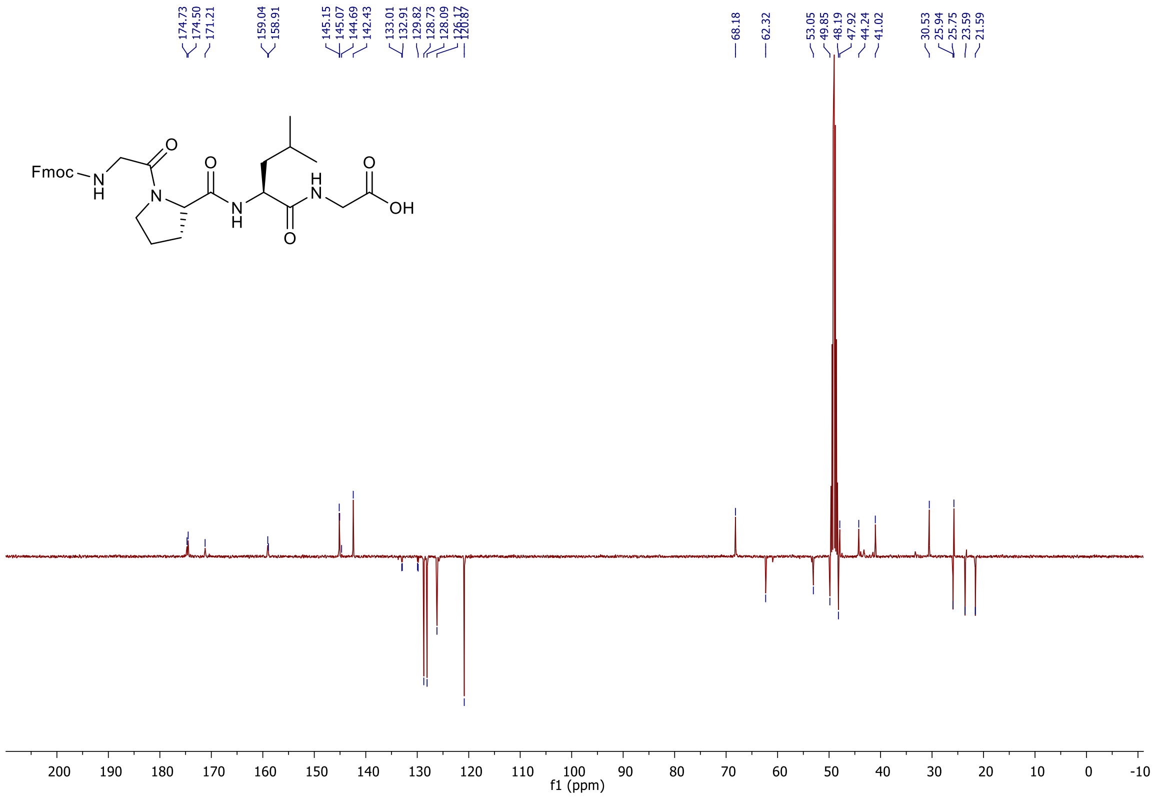

Figure S3.5 ^{1}H NMR spectrum (Acetone-d_6, 400 MHz) of Fmoc-GPLG-PAB-OH (4)

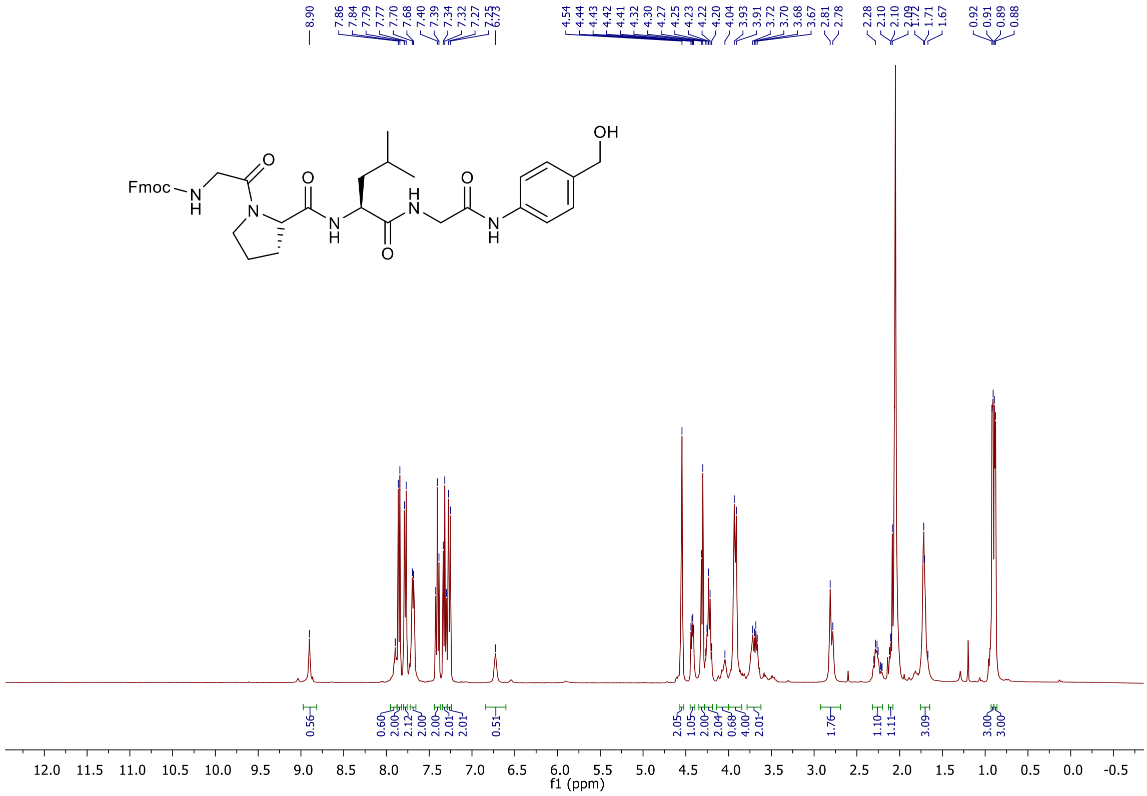

Figure S3.6 ^{13}C-JMOD NMR spectrum (Acetone-d_6, 101 MHz) of Fmoc-GPLG-PAB-OH (4)

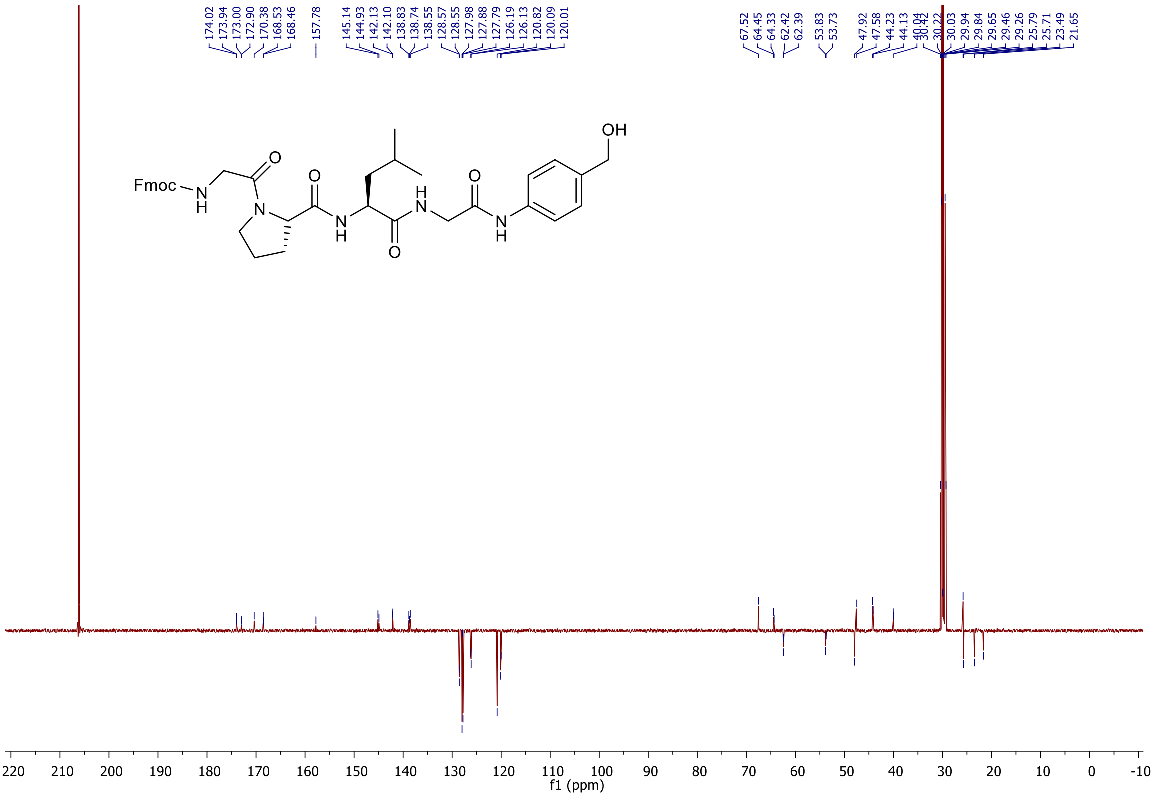

S4. Antiproliferative assay

Table S4.1 Antiproliferative effect of the conjugate and free drug MMAE in the presence of MMP-2 inhibitor (*cis*-9-octadecenoyl-*N*-hydroxylamide) on cancer cell lines with different integrin $\alpha_V\beta_3$ receptor level of expression after 72 h of continuous treatment.

	**IC_{50} [1] MMP-2 inhibitor + Treatment 72 h (nM)**		
	**U87MG ($\alpha_V\beta_3$ +)**	**SK-MEL-28 ($\alpha_V\beta_3$ +)**	**SK-OV-3 ($\alpha_V\beta_3$ +)**
***cyclo*[DKP-*iso*DGR]-PEG-4-GPLG-PABC-MMAE (2)**	7.4 ± 1.2	28.7 ± 1.7	3.2 ± 1.3
**MMAE**	0.3743 ± 0.1323	1.899 ± 0.7780	0.0334 ± 0.0119
**MMP-2 Inhibitor**	> 20000	> 20000	> 20000

[1] IC_{50} values (average ± SD).

S5. MMP-2 Degradation Assay

HPLC conditions for the MMP-2 cleavage studies

Samples were analyzed using analytical HPLC SHIMADZU LC-20AP equipped with diode array UV detector and LiChrosorb® RP-18 (5 μm) C18 column, flow rate: 1 mL/min., gradient: from 90% (H_2O + 0.1% TFA) / 10% (MeCN) to 40% (H_2O + 0.1% TFA) / 60% (MeCN) in 16 min. to 10% (H_2O + 0.1% TFA) / 90% (MeCN) in 9 min. to 10% (H_2O + 0.1% TFA) / 90% (MeCN) in 5 min. to 90% (H_2O + 0.1% TFA) / 10% (MeCN) in 5 min. to 90% (H_2O + 0.1% TFA) / 10% (MeCN) in 5 min.

Figure S5.1 H_2N-GPLG-PABC-MMAE (7) + buffer

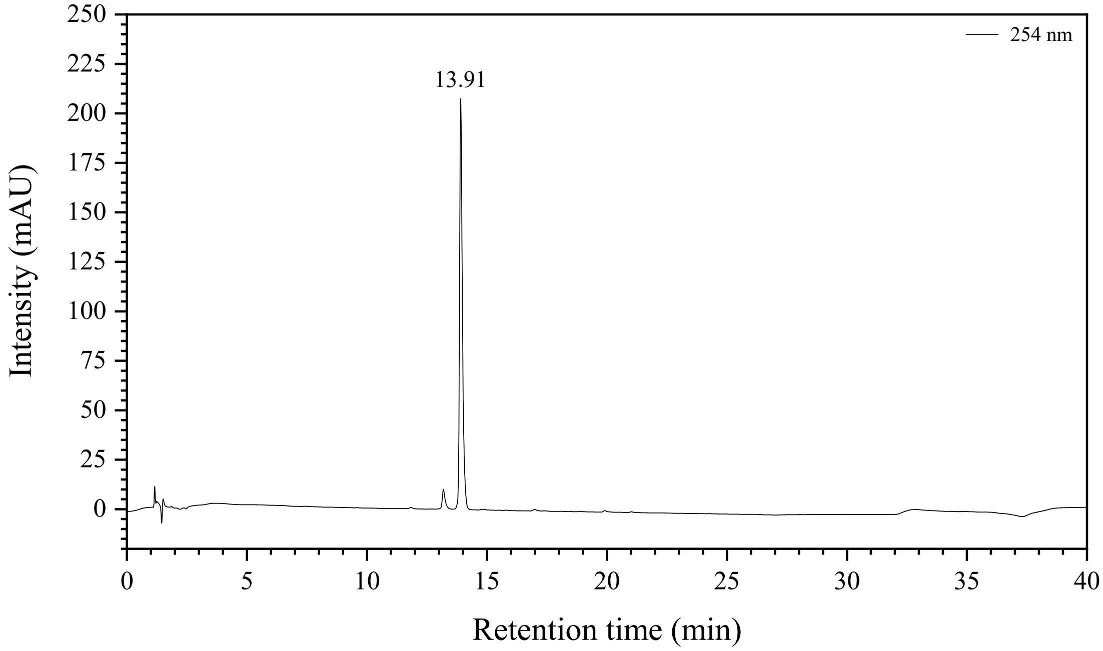

Figure S5.2 MMAE + buffer

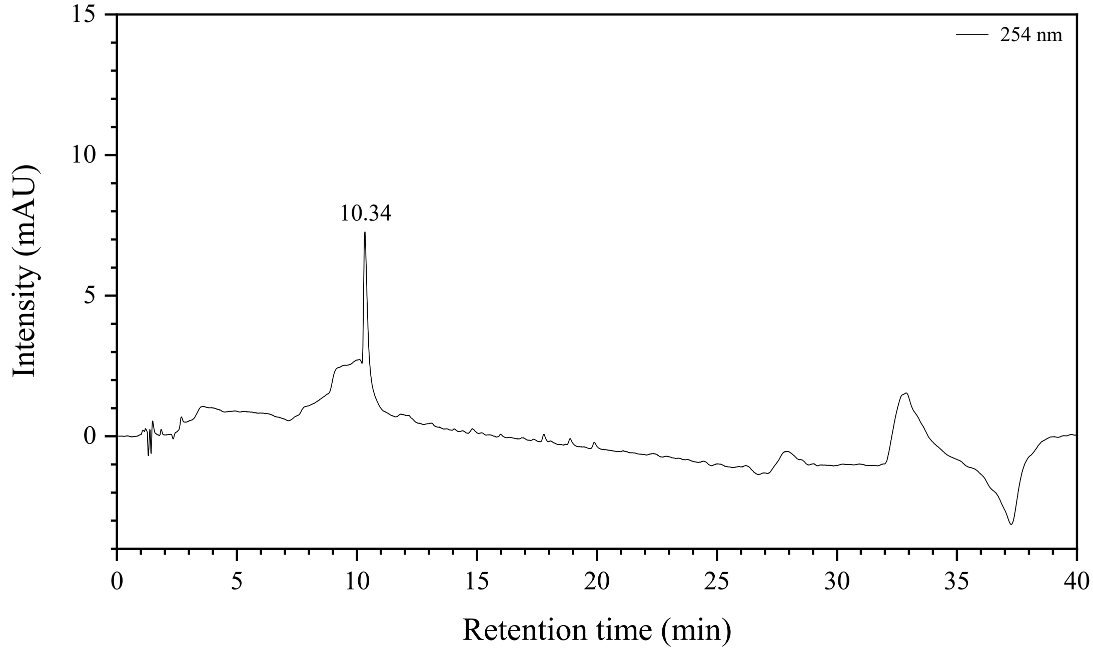

Figure S5.3 H_2N-GPLG-PABC-MMAE (7): MMP-2 enzymatic cleavage assay

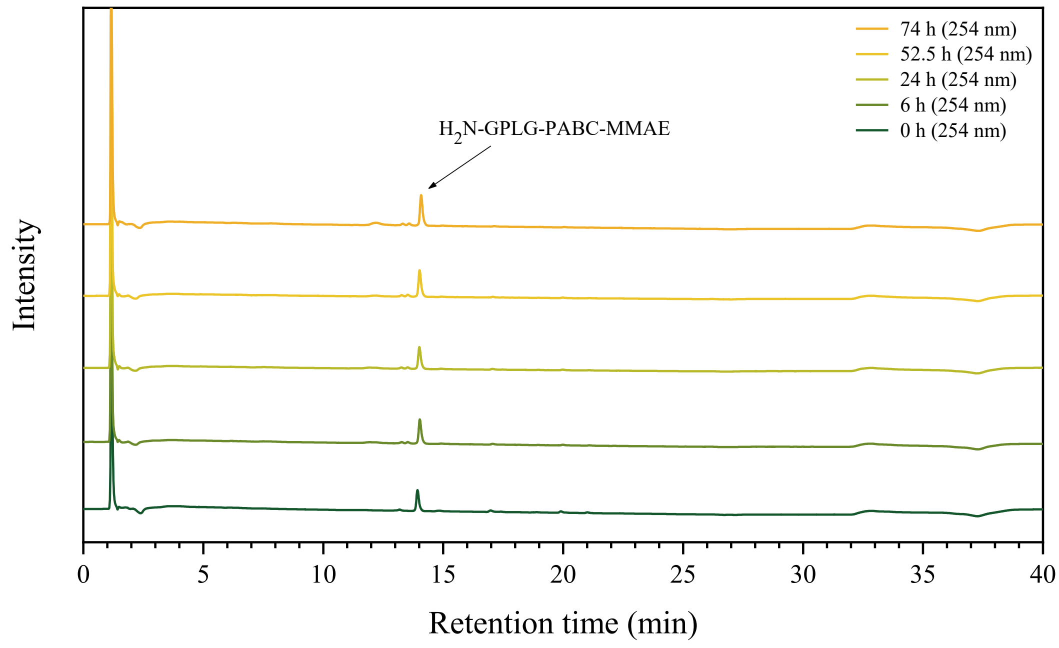

Figure S5.4 Fmoc-GPLGLAGG-OH + buffer

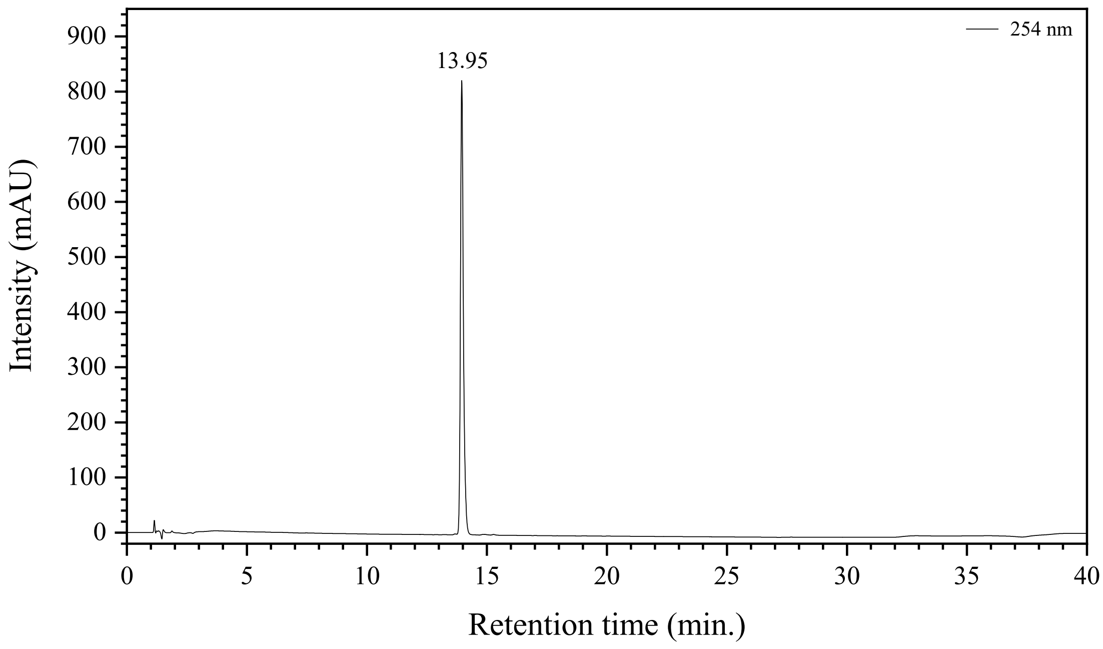

Figure S5.5 Fmoc-GPLG-OH (3) + buffer

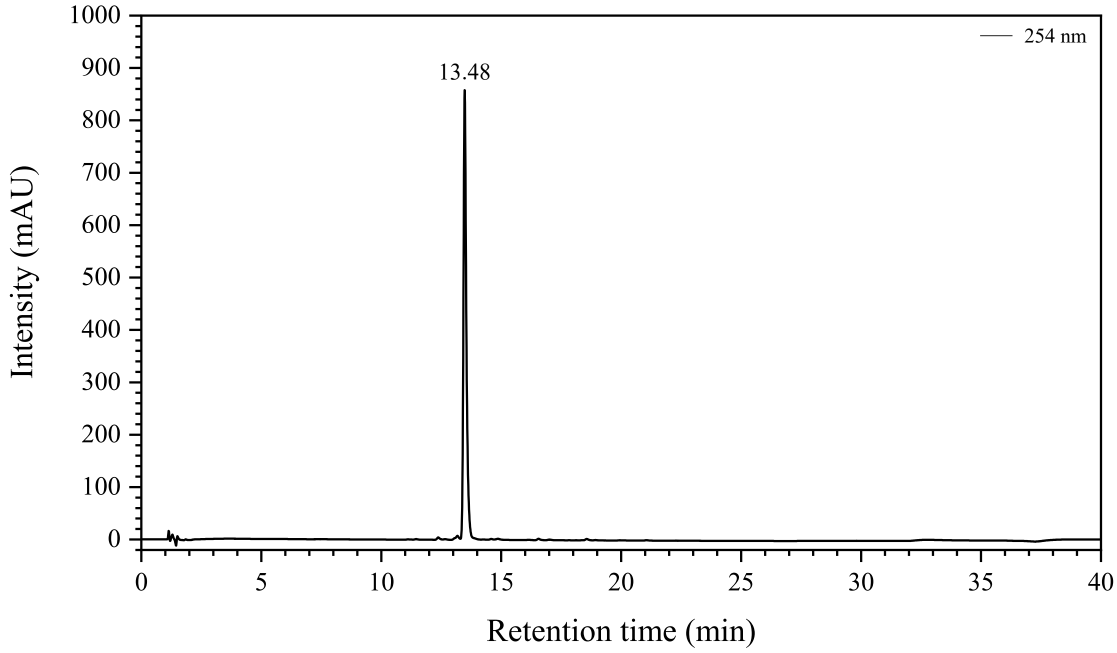

Figure S5.6 Fmoc-GPLGLAGG-OH: MMP-2 enzymatic cleavage assay

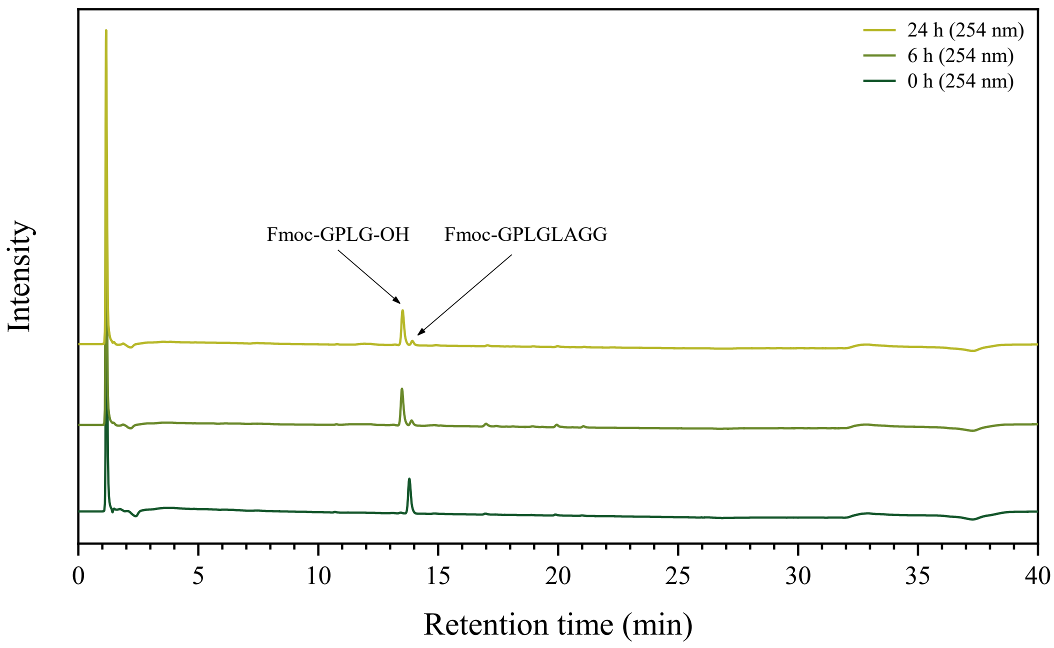

S6 Lysosomal Degradation assay

Figure S6.1 HPLC traces from the rat-liver lysosomal homogenate degradation assay

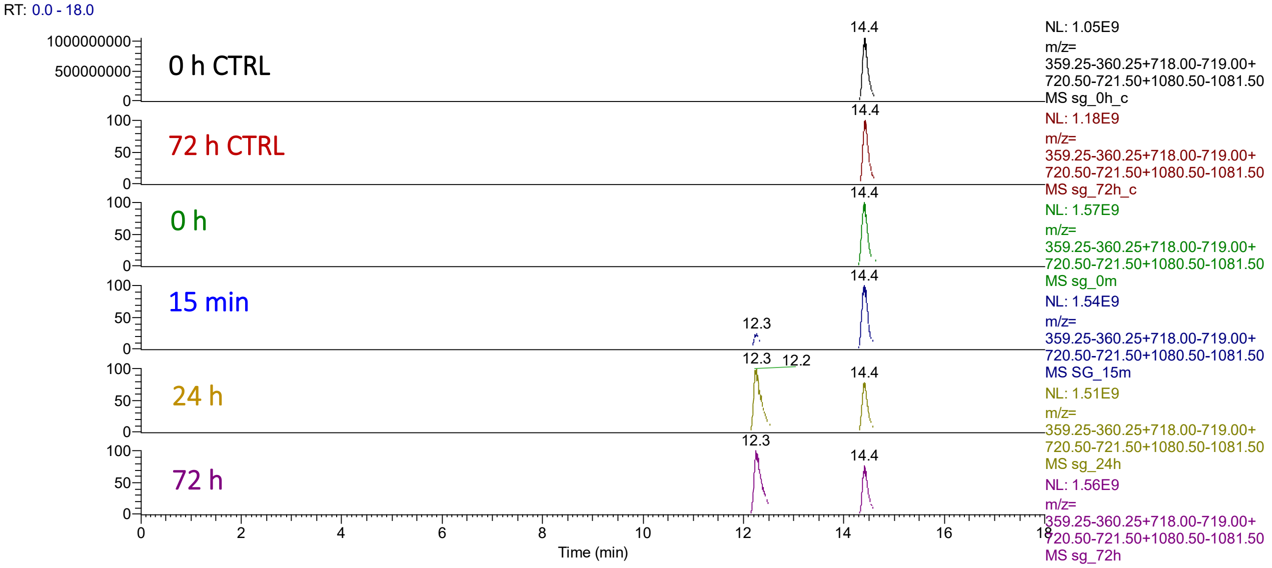

Figure S6.2 HRMS spectrum of the conjugate 2.

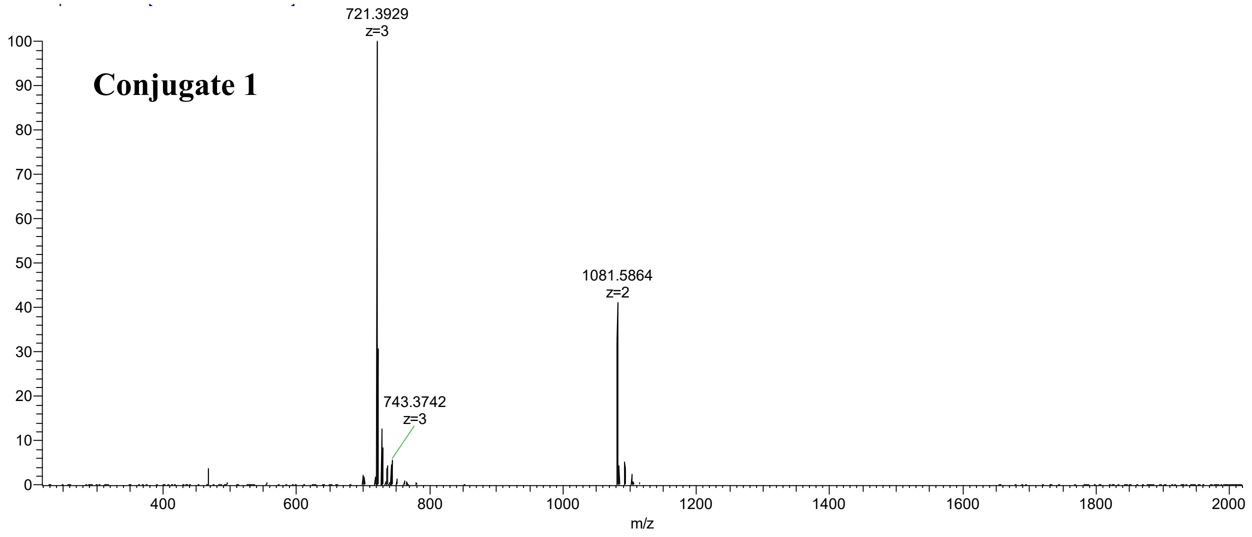

Figure S6.3 HRMS spectrum of the free MMAE.

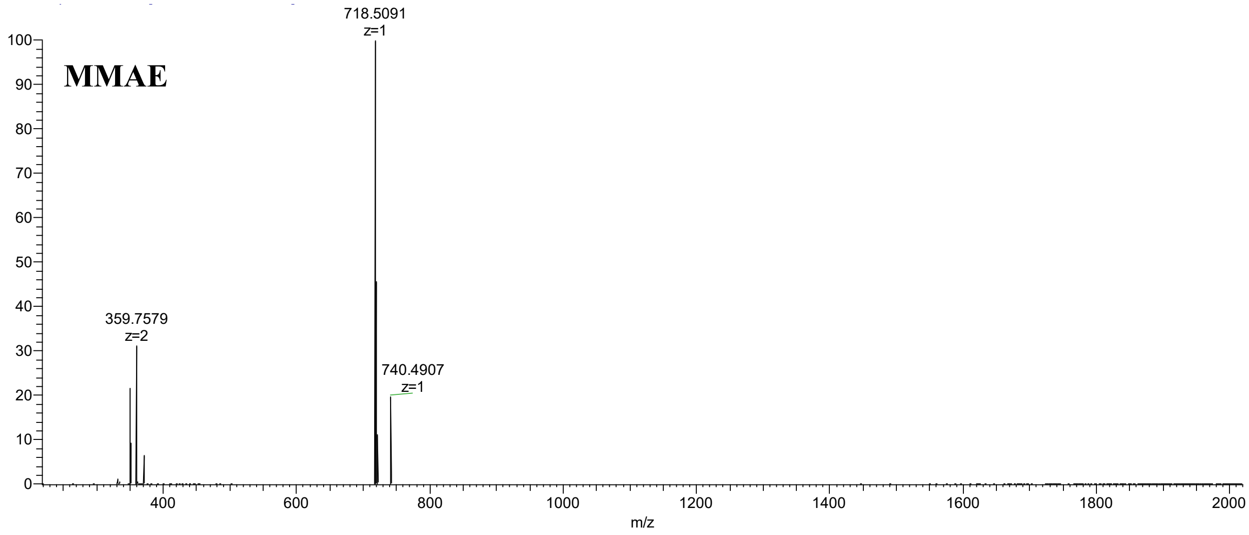

Table S6.1 Analytical details of the detected analytes

Conjugate **2**: Chemical Formula: $C_{104}H_{157}N_{23}O_{27}$		
m/z calculated for the analyte M	*m/z* measured for the analyte M	Mass error (ppm)
$[M+H]^{+}$=2161.1692	-	-
$[M+2H]^{2+}$=1081.0882	$[M+2H]^{2+}$=1081.0850	3.00
$[M+3H]^{3+}$=721.0613	$[M+3H]^{3+}$=721.0589	3.32
MMAE: Chemical Formula: $C_{39}H_{67}N_5O_7$		
m/z calculated for the analyte M	*m/z* measured for the analyte M	Mass error (ppm)
$[M+H]^{+}$=718.5113	$[M+H]^{+}$=718.5091	3.47
$[M+2H]^{2+}$=359.7593	$[M+2H]^{2+}$=359.7579	3.89

Supplementary References

[1] Bodero, L., Rivas, P.L., Korsak, B., Hechler, T., Pahl, A., Müller, C., Arosio, D., Pignataro, L., Gennari, C. and Piarulli, U. (2018). Synthesis and biological evaluation of RGD and *iso*DGR peptidomimetic-α-amanitin conjugates for tumor-targeting. *Beilstein J. Org. Chem.*, *14*(1), 407-415. Doi: 10.3762/bjoc.14.29
